# MiR-17-5p Inhibits TXNIP/NLRP3 Inflammasome Pathway and Suppresses Pancreatic β-Cell Pyroptosis in Diabetic Mice

**DOI:** 10.3389/fcvm.2021.768029

**Published:** 2021-11-22

**Authors:** Sijun Liu, Ge Tang, Fengqi Duan, Cheng Zeng, Jianfeng Gong, Yanming Chen, Hongmei Tan

**Affiliations:** ^1^Department of Pathophysiology, Zhongshan School of Medicine, Sun Yat-sen University, Guangzhou, China; ^2^Department of Endocrinology, The Third Affiliated Hospital of Sun Yat-sen University, Guangzhou, China

**Keywords:** diabetes mellitus, pancreatic β-cell, NLRP3 inflammasome, miR-17-5p, pyroptosis

## Abstract

**Objective:** Diabetes mellitus is a chronic progressive inflammatory metabolic disease with pancreatic β-cells dysfunction. The present study aimed to investigate whether miR-17-5p plays a protective effect on pancreatic β-cells function in diabetes mellitus (DM) mice and dissect the underlying mechanism.

**Methods:** C57BL/6J mice were randomly divided into control, DM, DM + Lentivirus negative control (LV-NC), and DM + Lenti-OE™ miR-17-5p (LV-miR-17-5) groups. DM was established by feeding a high-fat diet and intraperitoneal injection with streptozotocin (STZ) in mice. Blood glucose and glucose tolerance in circulation were measured. Meanwhile, the activation of nod-like receptor protein 3 (NLRP3) inflammasome, pancreas pyroptosis, and the expression of miR-17-5p and thioredoxin-interacting protein (TXNIP) were detected in the pancreas of DM mice. Pancreatic β-cell line INS-1 subjected to different concentrations of glucose was used in *in vitro* experiments.

**Results:** Compared with control mice, glucose tolerance deficit, elevated blood glucose level, and decreased pancreatic islet size, were presented in DM mice, which was associated with a downregulation of miR-17-5p. Importantly, exogenous miR-17-5p alleviated pancreas injury, and consequently improved glucose tolerance and decreased blood glucose in DM mice. *In vitro* experiments showed that high glucose decreased miR-17-5p expression and impaired insulin secretion in INS-1 cells. Mechanistically, miR-17-5p inhibited the expression of TXNIP and NLRP3 inflammasome activation, and thus decreased pancreatic β-cell pyroptosis.

**Conclusion:** Our results demonstrated that miR-17-5p improves glucose tolerance, and pancreatic β-cell function and inhibits TXNIP/NLRP3 inflammasome pathway-related pyroptosis in DM mice.

## Introduction

Diabetes mellitus is a serious public health concern globally, with a growing incidence caused by genetic or unhealthy lifestyle habits (1). Diabetes mellitus (DM) is characterized by long-term hyperglycemia resulting from the defect of insulin production and insulin resistance of insulin-responsive tissue and ultimately results in a series of chronic complications (2). Chronic low-grade inflammation is emerging as a key contributing factor to DM (3). However, the detailed mechanism of inflammatory-related dysglycemia and defect of insulin secretion in DM remains largely unexplored.

Nod-like receptor protein 3 (NLRP3) inflammasome has a critically important role in the regulation of inflammation in the development of DM (4). NLRP3 responds to a variety of stimulators, such as toxins, pathogens, metabolites, crystalline substances, nucleic acids, and ATP, thereby initiating the assembly of the NLRP3 inflammasome and facilitating the autocleavage and maturation of precursors of caspase-1 (5). And activated caspase-1 cleaves gasdermin D (GSDMD) to generate an N-terminal GSDMD fragment (GSDMD-NT). The GSDMD-NT targets the plasma membrane and results in the formation of a cell pore, cell tumid, and lysis, which initiates a programmed death process referred to as pyroptosis (6, 7). In patients with DM, NLRP3 inflammasome is activated, and successive proinflammatory mediators also significantly elevate (8). It was reported that thioredoxin-interacting protein (TXNIP), a multifunctional and inducible protein, regulates redox homeostasis in the cells (9, 10). Increased expression of TXNIP functions as a major role in β cell failure and dysfunction during diabetes development (11). Meanwhile, TXNIP exacerbates NLRP3 inflammasome activation and provokes oxidative stress (12). Therefore, inhibiting TXNIP/NLRP3 inflammasome pathway becomes a new therapeutic strategy in DM induced by an excessive inflammatory response.

MicroRNAs are a kind of small endogenous RNAs that bind messenger RNAs (mRNA) to regulate translation and stability of mRNA, and microRNAs play a critical role in the regulation of mechanisms operating in various biologic functions such as NLRP3 inflammasome activation and pyroptosis (4, 13, 14). Some microRNAs have critical roles in insulin secretion, pancreatic β-cell development and function, and the pathogenesis of DM (15). MiR-17-5p is a member of the miR-17-92 cluster, and it was confirmed that miR-17-5p was downregulated in mice embryos from diabetic dams (16). MiR-17-92 cluster is the first group of microRNAs to be indicated in the regulation of the development of multiple tissues and is indispensable for cell proliferation and cell cycle. Moreover, some researchers implicated that miR-17-5p was involved in the regelation of the progression of multiple cancer types and the exertion of a diverse function in the development and survival of pancreatic β-cell (17, 18). However, the role of miR-17-5p in the pathological state of DM is still unclear. The present study intended to explore the potential effect of miR-17-5p on pancreatic β-cell pyroptosis in a mouse model of DM and dissect the underlying mechanism.

## Methods

### Cell Culture and Cell Transfection

Rat INS-1 pancreatic β-cell line (ATCC, Manassas, VA, USA) were grown in Roswell Park Memorial Institute 1640 medium supplemented with 10% fetal bovine serum (Gibco, USA), 2.5 mmol/L mercaptoethanols, 10 mmol/L HEPES, 100 U/ml penicillin-streptomycin, and 11.1 mmol/L glucose, and incubated at 37°C in a 5% carbon dioxide incubator. INS-1 cells were transfected with miR-17-5p mimic (RiboBio Co., Ltd., Guangzhou, China) according to standard protocol in the presence of different concentrations of glucose (5 and 25 mmol/L). In some experiments, INS-1 cells were treated, respectively, with different concentrations of glucose (5, 25, and 50 mmol/L) for 72 h. To investigate whether miR-17-5p inhibited the expression of TXNIP, INS-1 cells were transfected with miR-17-5p mimic or miR-17-5p inhibitor in the presence of high glucose (25 mmol/L).

### Glucose-Stimulated Insulin Secretion Assay (GSIS)

The INS-1 cells were transfected with negative control or miR-17-5p mimic as described above, respectively, in normal (5 mmol/L) or high glucose (25 mmol/L) conditions for 72 h. Then, cells were preincubated with Krebs-Ringer bicarbonate HEPES Buffer (KRBH) (Gibco, USA) containing 2.8 mmol/L glucose for 2 h. For insulin stimulation, cells were transferred to a new KRBH buffer with 16.7 mmol/L of glucose and incubated for 1 h at 37°C. Insulin concentration was determined relying on an insulin Elisa kit (Cloud-Clone Corp, Wuhan, China) in the supernatant.

### Flow Cytometry Analysis for Cell Pyroptosis

To identify pyroptosis in INS-1 cells, cells were treated with high glucose (25 mmol/L) in the presence or absence of miR-17-5p mimic for 72 h, or cells were incubated with normal glucose (5 mmol/L) condition for 72 h. The cells were incubated with active caspase-1 and 7-aminoactinomycin D (7-AAD) for 15 min in 37°C, then analyzed by Flow cytometry (BECHMAN COULTER, Cytoflex, Indianapolis, IN, USA). Active caspase-1 was analyzed using the FAM-YVAD-FMK caspase-1 detection kit (Cell technology, Mountain View, CA, USA). 7-AAD labeled the DNA of cells because of loss of membrane integrity. Active caspase-1^+^/7-AAD^+^ cells were defined as pyroptotic cells.

### Animal Protocol

We obtained the C57BL/6J mice (8 weeks, male, 20–25 g) from Vital River Laboratory Animal Technology Co., Ltd. (Beijing, China). Mice were housed in specific pathogen-free conditions around 24°C at the laboratory animal center of Sun Yat-sen university under a 12-h light/dark cycle with free access to water and food. All animal protocols were approved by ethic committees of Sun Yat-sen University and all experiments complied with the Guide for the Care and Use of Laboratory Animals published by the US National Institutes of Health (No. 85–23, revised 1996). Mice were randomly divided into four groups: control group, DM group, DM + Lentivirus negative control (LV-NC) group, and DM + Lenti-OE™ miR-17-5p (LV-miR-17-5) group (*n* = 8 for each group). Specifically, control mice were fed with a standard chow diet [protein, 26.5%; fat, 14%; carbohydrates, 59.5%; (2019) 05073, Guangdong Medical Laboratory Animal Center, Guangdong, China]. The other mice were fed with high-fat diet (protein, 20%; fat, 39.9%; carbohydrates, 40%; MD12017, Mediscience Ltd., Jiangsu, China) for 8 weeks. Mice were injected intraperitoneally with streptozotocin (STZ) (Sigma Aldrich, St. Louis, MO, USA) (100 mg/kg/d) for a 5-day schedule to induce DM. Blood glucose level over 16.6 mmol/L was considered as DM. After the DM model was established, lentivirus (Genechem Co., Ltd., Shanghai, China) was transfected into the tail vein at a dose of 1 × 10^7^ U. To ensure the stability and function of miR-17-5p in mice, miR-17-5p was transfected into mice every 4 weeks. Additionally, blood glucose was measured every 4 weeks. Then, 20 weeks after the last injection, all mice were sacrificed by exsanguination after anesthesia with pentobarbital (80 mg/kg body weight). Blood samples and the pancreatic tissue of mice were collected and stored at −80°C for further analysis.

### Blood Glucose Measurement and Intraperitoneal Glucose Tolerance Test (IPGTT)

Blood glucose level was determined by OneTouch Ultra2 Glucose Monitors (LifeScan, Milpitas, CA, USA) every 4 weeks after STZ injection from tail vein blood samples. For IPGTT tests, mice were injected intraperitoneally with glucose solution (1 g/kg), and blood glucose level was measured as mentioned above at 15, 30, 60, and 120 min after injection. Plot the blood glucose concentration curve. Additionally, the area under the curve (AUC) for IPGTT was calculated and analyzed as previously reported (19).

### Immunohistochemistry

Pancreatic tissue from mice was dissected and processed into paraffin blocks after fixation with 4% formaldehyde. Then, the tissue was cut into 5 μm sections. After deparaffinization and retrieval of antigen, sections were stained with anti-insulin antibody (1:200 dilution, R&D System, Minneapolis, MN, USA). The sections were incubated with anti-rat IgG as secondary antibodies and subsequently with 3,3′-diaminobenzidine as chromogen the next day. Images were acquired by using Leica DM2500B in 10 × objective magnification. Image-Pro Plus 6 software (Media Cybernetics, Rockville, Md) was used to analyze and calculate the area of insulin-positive area and the overall area. For the islet size quantitation, the same region of the pancreas was selected for immunohistochemistry. Additionally, four different slides were quantitated to create an average for each mouse.

### Quantitative Real-Time Polymerase Chain Reaction (qRT-PCR)

The total RNA of INS-1 cells or pancreatic tissue was extracted using a Mini-BEST Universal RNA Extraction kit (TaKaRa, Kyoto, Japan) according to the protocols of the manufacturer. The concentration and purity of RNA were determined with an ultraviolet spectrophotometer (Thermo Fisher Scientific, CA, USA). RNA was then reverse transcribed into cDNA using a Transcriptor First Strand cDNA Synthesis Kit (Roche Diagnostics, Germany). Then, qRT-PCR were carried out with Real-Time PCR System (Bio-Rad, CA, USA) using the SYBR Green Master Mix kit (Roche Diagnostics, Germany). The primers used were as follows: miR-17-5p: CAAAGUGCUUACAGUGCAGGUAG.

### Western Blot Analysis

Protein from pancreatic tissue and INS-1 cells lysates were collected. Western blot procedures were processed as standard protocol with specific antibodies against TXNIP (1:1,000, Abcam, UK), GSDMD (1:1,000, Abclonal, USA), caspase-1 (1:1,000, Abclonal, USA), NLRP3 (1:1,000, Adipogen, USA), and β-actin (1:5,000, Sigma, USA). The blots were visualized by ChemiDoc™ Touch Imaging System (Bio-Rad, CA, USA). Western blot image was analyzed and quantified by using Image J software (NIH, Bethesda, MD, USA). Relative expression of the protein was normalized with β-actin expression.

### Statistical Analysis

The results were presented as *M* ± *SD*. All *in vitro* experiments were completely randomized designed and repeated in triplicate. Data were analyzed using GraphPad Prism 6 software (CA, USA). Statistical comparison among multiple groups was carried out by one-way ANOVA followed by an LSD test. *P* < 0.05 was considered statistically significant.

## Results

### The Effect of High Glucose on Expression of miR-17-5p, TXNIP, and GSIS in INS-1 Cells

**Figure 1:**
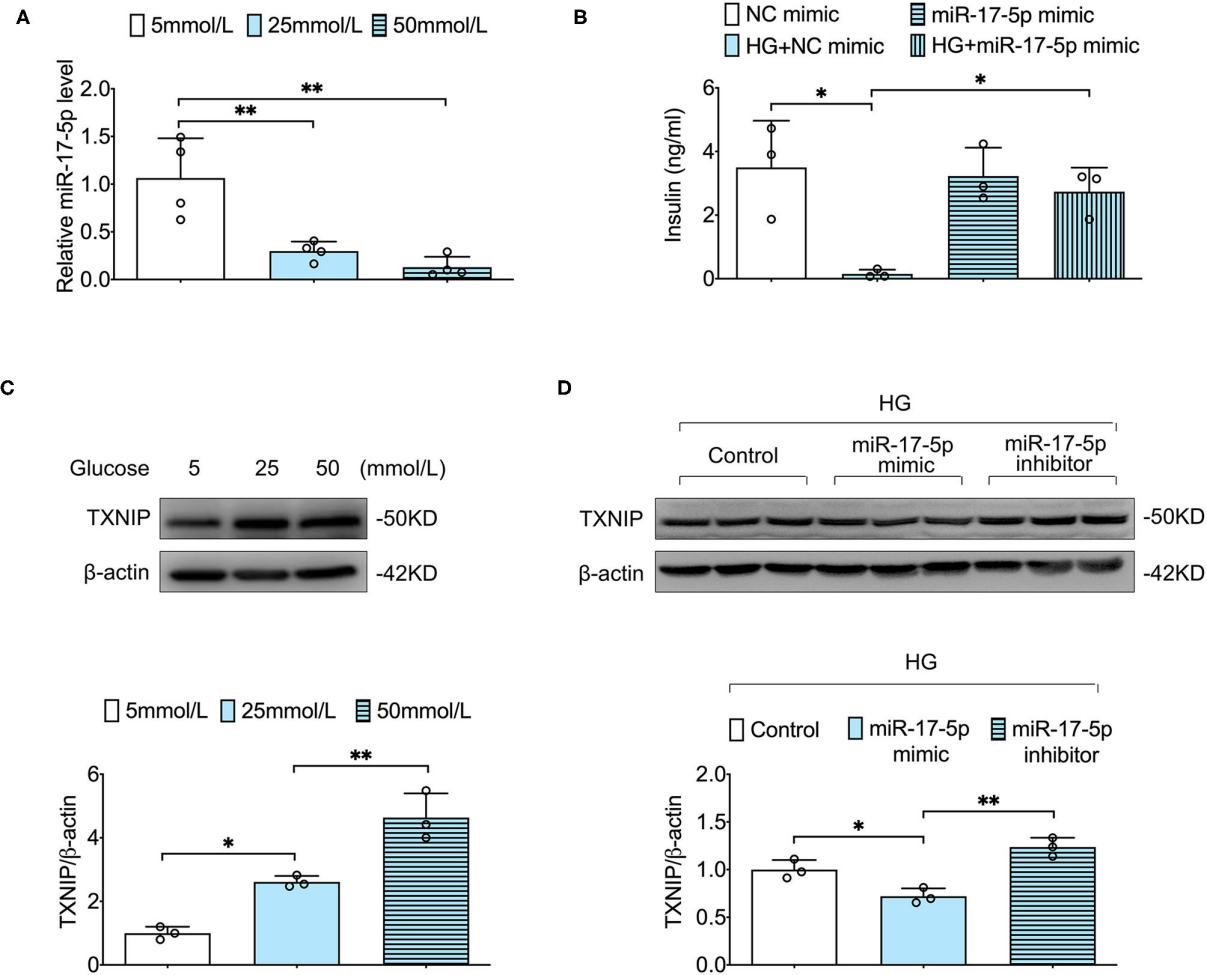
The effect of high glucose on expression of miR-17-5p, TXNIP, and GSIS in INS-1 cells. **(A)** The relative levels of miR-17-5p under different concentrations of glucose were analyzed by qRT-PCR. **(B)** Effects of high glucose and miR-17-5p on GSIS. **(C)** Representative blots and quantitative analysis showing the expression of TXNIP in INS-1 cells treated with different concentrations of glucose. **(D)** The effects of miR-17-5p on the expression of TXNIP in INS-1 cells. Control: 5 mmol/L; HG (high glucose): 25 mmol/L. ^*^p < 0.05 and ^**^p < 0.01.

To determine the effect of different concentrations of glucose (5, 25, and 50 mmol/L) on INS-1 cells, we examined the expression level of miR-17-5p by using qRT-PCR in different groups. When INS-1 cells were incubated in high glucose (25 and 50 mmol/L), the expression of miR-17-5p was significantly down-regulated, demonstrating that high glucose resulted in decreased expression of miR-17-5p in a dose-dependent model (Figure 1A). The synthesis and secretion of insulin by pancreatic β-cell were essential indicators of pancreatic β-cell function. To determine whether miR-17-5p affected the insulin secretion function in high glucose conditions, we detected the concentration of insulin in the presence or absence of miR-17-5p mimicked by GSIS in INS-1 cells. Our data showed that cells treated with high glucose secreted less insulin, and miR-17-5p mimic rescued insulin secretion function of INS-1 cells (Figure 1B). TXNIP is an oxidative stress-sensitive activator in cellular redox balance and is involved in the progression of diabetes (20), and we found that high glucose increased expression of TXNIP in a glucose dose-dependent manner (Figure 1C). Importantly, high glucose-induced TXNIP expression was inhibited by the administration of miR-17-5p mimic, while enhanced by a miR-17-5p inhibitor (Figure 1D). Collectively, these results indicated that high glucose decreased the expression of miR-17-5p, increased the expression of TXNIP, and impaired GSIS in INS-1 cells. The expression of TXNIP was regulated by miR-17-5p mimic.

### MiR-17-5p Mimicked the Suppressed High Glucose-Induced Expression of TXNIP, NLRP3 Inflammasome Activation in INS-1 Cells

**Figure 2:**
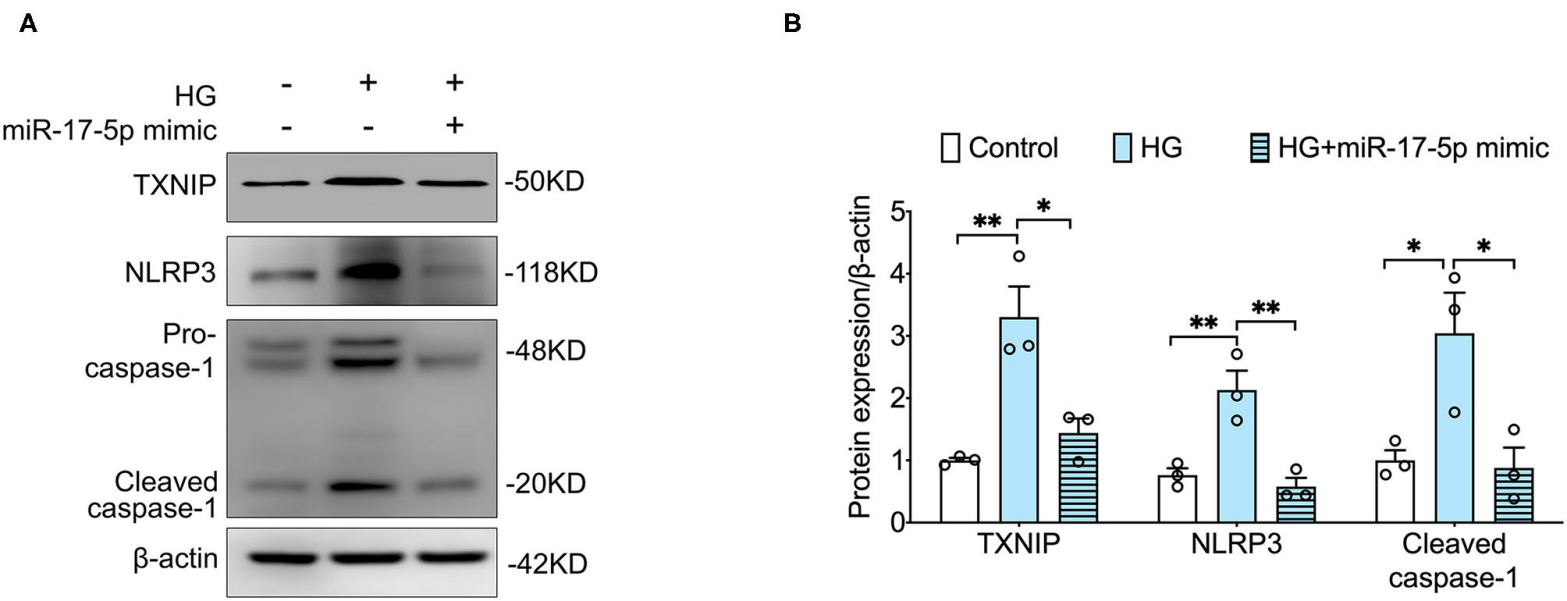
MiR-17-5p mimic suppressed high glucose-induced hyperactivated NLRP3 inflammasome and the expression of TXNIP in INS-1 cells. **(A,B)** Representative blots and quantitative analysis showing the expression of NLRP3, the cleavage of caspase-1, and TXNIP. Control: 5 mmol/L; HG (high glucose): 25 mmol/L. ^*^p < 0.05 and ^**^p < 0.01.

Given that TXNIP played a central role in NLRP3 inflammasome activation (21), we determined to detect whether NLRP3 inflammasome was activated in INS-1 cells. Our data showed that high glucose notably increased the expression of TXNIP and NLRP3 and the cleavage of caspase-1 in INS-1 cells, indicating that high glucose activated TXNIP/NLPR3 inflammasome pathway in INS-1 cells. Importantly, the expression of TXNIP, NLRP3, cleaved caspase-1 was suppressed by a miR-17-5p mimic in high glucose-treated INS-1 cells (Figures 2A,B), suggesting that the protective effect of miR-17-5p is related to the TXNIP/NLRP3 inflammasome pathway.

### MiR-17-5p Mimicked the Inhibiting High Glucose-Induced Pyroptosis in INS-1 Cells

**Figure 3:**
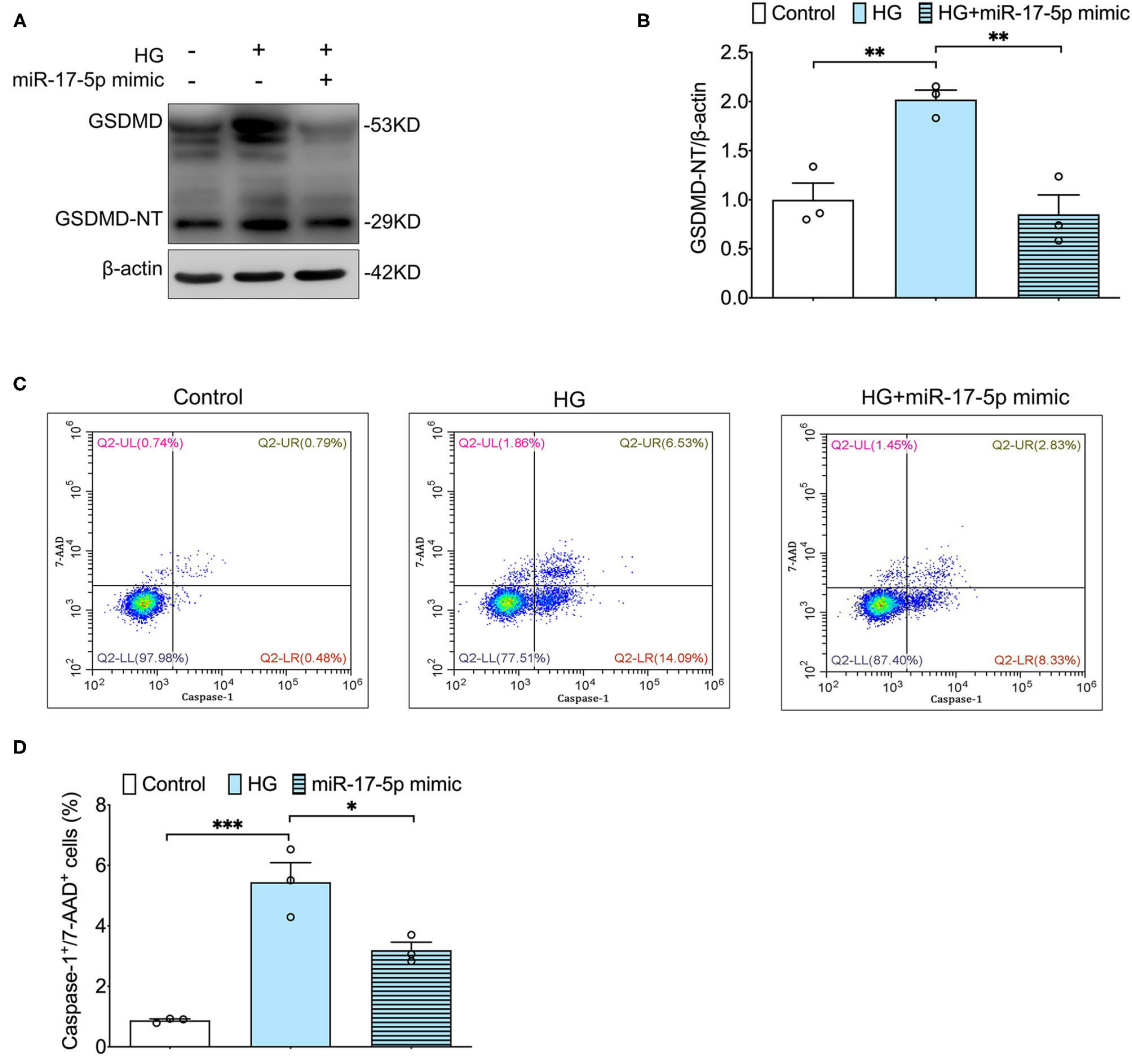
MiR-17-5p mimic inhibited high glucose-induced pyroptosis in INS-1 cells. **(A,B)** Representative blots and quantitative analysis showing the expression of cleavage of GSDMD in INS-1 cells. **(C,D)** Representative flow cytometric dot plots and the corresponding quantification showing active caspase-1+/7-AAD+ cells as pyroptosis population in INS-1 cell cultured with different concentrations of glucose. Control: 5 mmol/L; HG (high glucose): 25 mmol/L. ^*^p < 0.05, ^**^p < 0.01, and ^***^p < 0.001.

Furthermore, we determined whether miR-17-5p suppressed pyroptosis based on TXNIP/NLRP3 inflammasome pathway in INS-1 Cells. Our results showed that the cleavage of GSDMD, an important indicator for pyroptosis, was inhibited by a miR-17-5p mimic in INS-1 cells, indicating miR-17-5p inhibited high glucose-induced pyroptosis (Figures 3A,B). Then, double staining of active caspase-1 and 7-AAD were performed to quantify pyroptotic cells. An increased population of pyroptotic cells (active caspase-1^+^/7-AAD^+^ cells) was observed in high glucose treatment cells, which was reduced by a miR-17-5p mimic in INS-1 cells (Figures 3C,D). These results suggested miR-17-5p mimic suppressed high glucose-induced pyroptosis in INS-1 cells.

### The Effect of Overexpression of miR-17-5p on Blood Glucose, Glucose Tolerance, and Pancreatic Islet Size in DM Mice

**Figure 4:**
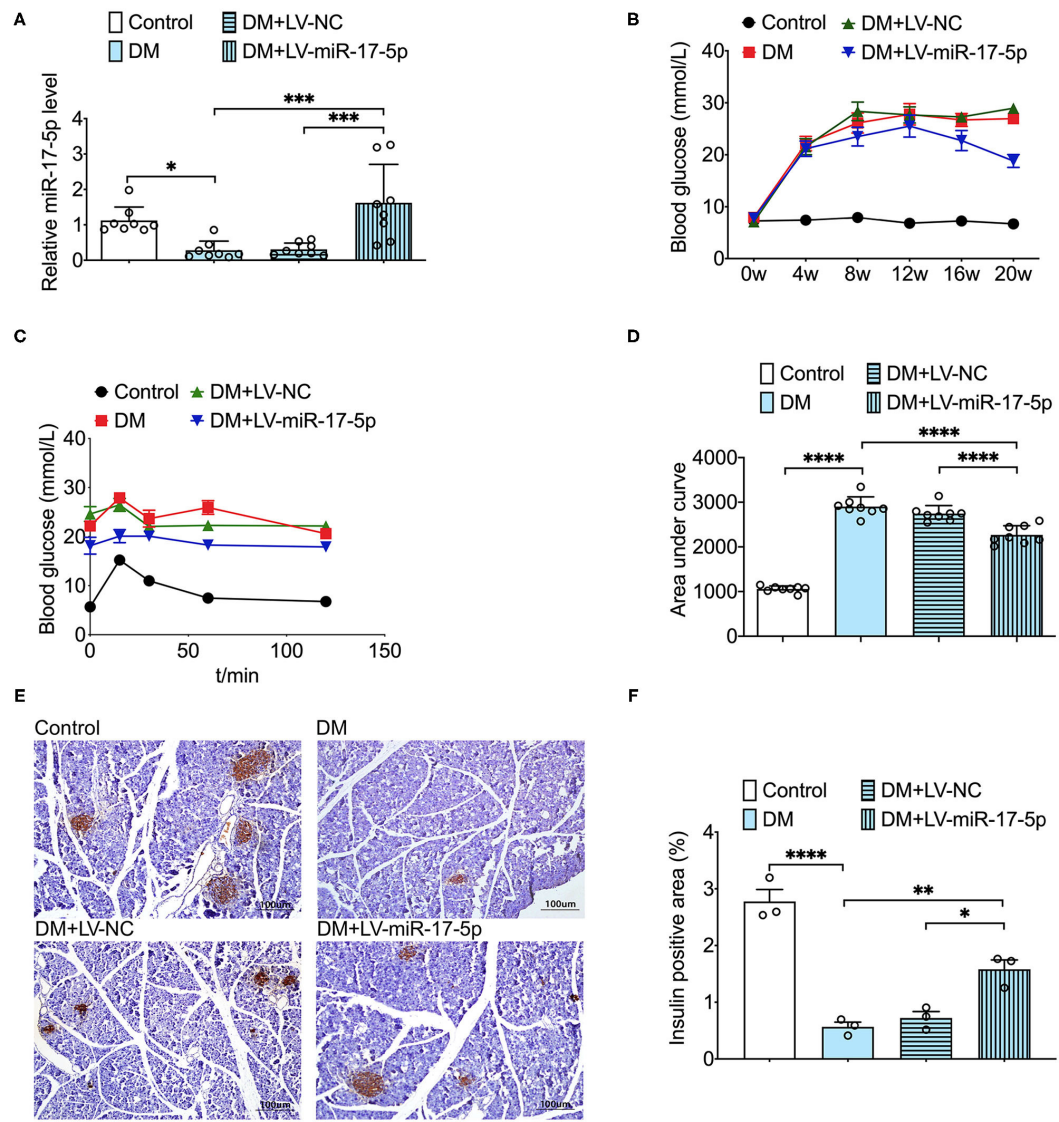
Overexpression of miR-17-5p decreased the levels of blood glucose, improved glucose tolerance, and increased the pancreatic islet size in the DM mice. **(A)** The level of miR-17-5p in mice pancreatic tissue was determined by qRT-PCR (*n* = 8). **(B)** Effects of miR-17-5p on the blood glucose levels in DM mice (*n* = 8). **(C,D)** Effect of miR-17-5p on glucose tolerance in DM mice (*n* = 8). **(E,F)** Pancreatic sections were immunohistochemically stained for anti-insulin (dark brown). All slides were counterstained with hematoxylin and imaged at ×10 objective magnification (*n* = 3). ^*^p < 0.05, ^**^p < 0.01, ^***^p < 0.001, and ^****^p < 0.0001.

To further investigate the protective effect of miR-17-5p in DM mice, lentivirus technology was carried out to increase the expression of miR-17-5p in DM mice. Besides, we further found the expression of miR-17-5p was significantly down-regulated in DM mice. Then, we examined the blood glucose in circulation and found that overexpression of miR-17-5p remarkably lowered the high blood glucose at the 12th week after the first injection, and the blood glucose levels were significantly lower than that in the DM mice at the end of the experiment. Moreover, when DM mice were transfected with miR-17-5p, the calculated AUC for IPGTT was reduced significantly compared with the DM mice, suggesting that miR-17-5p improved glucose tolerance (Figures 4A–D). Additionally, the immunohistochemical study showed that the pancreatic islet size has a significant reduction in the DM mice. After increased the expression of miR-17-5p, pancreatic islet size was increased significantly in DM mice (Figures 4E,F). These findings suggested miR-17-5p could mitigate high glucose, improve the glucose tolerance in circulation and display a markedly protective effect on normalizing the pancreatic β-cell function in DM mice.

### Overexpression of miR-17-5p Suppressed TXNIP/NLRP3 Inflammasome Pathway and Pyroptosis in DM Mice

**Figure 5:**
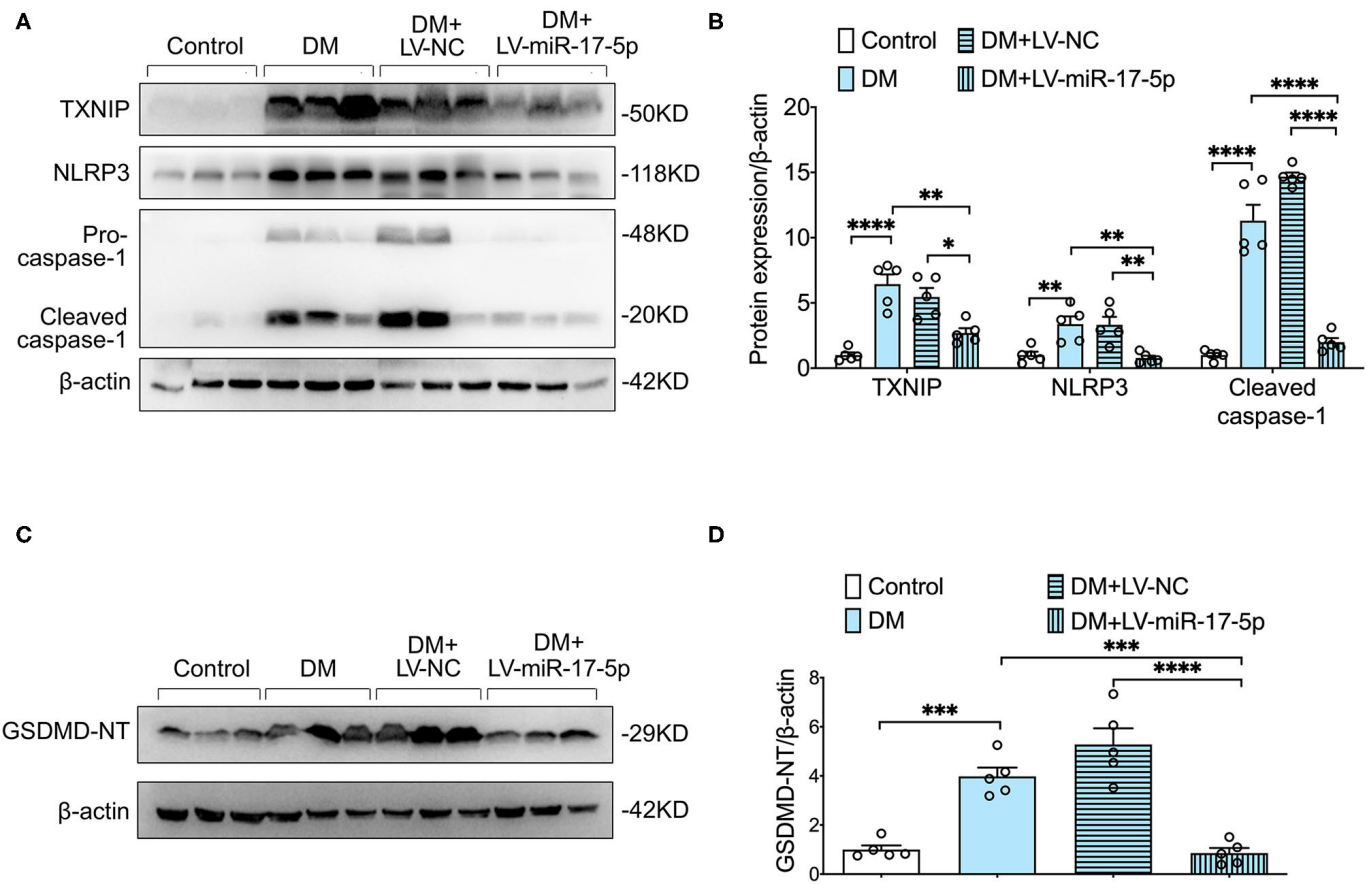
Overexpression of miR-17-5p ameliorated NLRP3 inflammasome-mediated pyroptosis, and suppressed TXNIP expression in DM. **(A,B)** Representative blots and quantitative analysis showing the expression of NLRP3, the cleavage of caspase-1, and TXNIP. **(C,D)** GSDMD. ^*^p < 0.05, ^**^p < 0.01, ^***^p < 0.001, and ^****^p < 0.0001.

We further examined whether overexpression of miR-17-5p inhibited the expression of TXNIP and NLRP3 inflammasome activation in the pancreas of DM mice. Enhanced expression of the expression of TXNIP, NLRP3, and cleaved caspase-1 was presented in DM mice compared with control mice, indicating the TXNIP/NLRP3 inflammasome pathway was relevant to the progression of DM mice. Importantly, both increased expression of TXNIP and hyperactivated NLRP3 inflammasome were inhibited by the exogenous miR-17-5p in DM mice (Figures 5A,B). In addition, cleavage of GSDMD was increased significantly in DM mice and was also suppressed by the up-regulation of miR-17-5p (Figures 5C,D). Consistent with *in vitro* results, miR-17-5p inhibited TXNIP/NLRP3 inflammasome pathway and pyroptosis in DM mice.

## Discussion

In the current study, we demonstrated that miR-17-5p inhibits TXNIP/NLRP3 inflammasome pathway, suppresses pancreatic β-cell pyroptosis, and improves pancreatic β-cell function in diabetic mice. Our data proved that miR-17-5p could serve as a promising therapeutical strategy in excessive inflammatory response-induced pancreas injury in DM.

It was reported that miR-17-5p plays different effects on many complications. A recent study demonstrated that miR-17-5p acted as a major culprit for promoting breast cancer metastasis and was enhanced in colorectal cancer (22, 23). Despite the significance of miR-17-5p in some cancers, very few studies have focused on the effects of miR-17-5p in metabolic disease. Klöting et al. reported that the expression of miR-17-5p significantly decreased in obese non-diabetic patients (24). In the present study, we further identified that the expression of miR-17-5p was decreased in the pancreas of DM mice and INS-1 cells cultured with high glucose. Moreover, overexpression of miR-17-5p not only decreased blood glucose but also significantly improved glucose tolerance in DM mice.

Diabetes mellitus involving a defect in insulin secretion causes severe complications (21). Pancreatic β-cell plays a vital role in the control of blood glucose homeostasis. At present, a growing number of studies showed that microRNAs did not only affect the differentiation and proliferation of β-cell but also affected the synthesis and secretion of insulin (25, 26). The study of Hong et al. reported that proinflammatory cytokine interferon γ reduced the expression of miR-17, in the case of endoplasmic reticulum stress in INS-1 cells (27). The GSIS test was often used to determine the insulin release function of pancreatic β-cell. Prolonged elevated glucose stimulation could result in β cell dysfunction and β cell mass reduction (28). In accordance with the previously reported, INS-1 cells exposed to high-glucose conditions showed significantly decreased insulin secretory function. However, miR-17-5p mimic potentiated the GSIS in INS-1 cells in response to high glucose. To further certificate the protective ability of miR-17-5p on pancreatic β-cell, miR-17-5p was overexpressed in DM mice. We disclosed that the up-regulation of miR-17-5p significantly increased the pancreatic islet size in pancreatic tissue. Herein, we revealed that miR-17-5p might be a new promising therapeutical strategy for β-cell protection in DM.

Hyperglycemia exacerbates the development of inflammation, and chronic inflammation can lead to pancreas injury (29). Recently research demonstrated that TXNIP played important role in glucose metabolism, and high glucose condition up-regulated the expression of TXNIP, respectively, in cells and diabetes rats (30, 31). Moreover, TXNIP knockdown or deletion improved glucose tolerance and pancreatic β-cell function in mice (21). Consistent with our previous study (32), we found that increased expression of TXNIP and hyperactivated NLRP3 inflammasome were observed in diabetic mice, indicating that TXNIP/NLRP3 inflammasome pathway plays a central role in the pathophysiology of DM. Bioinformatic analysis indicated that miR-17 regulates TXNIP expression by interacting with the two conserved binding sites in the TXNIP 3′-UTR (16, 33). Moreover, some researchers demonstrated that the anti-inflammatory effect of miR-17-5p was associated with the inhibition of TXNIP/NLRP3 inflammasome in the brain (34, 35). However, the protective effect of miR-17-5p on DM is unclear. Thus, we tried to find the bridge between miR-17-5p and TXNIP/NLRP3 inflammasome pathway in DM. According to our works, high-abundance of TXNIP and NLRP3 inflammasome activation were validated in DM mice and INS-1 cells, and importantly, which were reversed by miR-17-5p. Moreover, we discovered miR-17-5p mimic inhibited high glucose-induced expression of TXNIP, while miR-17-5p inhibitor promoted TXNIP expression in INS-1 cells. These results suggested that miR-17-5p acted as an upstream factor in regulating TXNIP/NLRP3 inflammasome pathway in DM.

Pyroptosis is a type of GSDMD-mediated programmed cell death. The NLRP3 inflammasome, a vital activator of proptosis, triggers the release of GSDMD-NT. The GSDMD-NT is the effector molecule of pyroptosis, which forms membrane pores and then induces the secretion of proinflammatory cytokines (36, 37). Accumulating evidence highlights a central role for pyroptosis in the pathogenesis of DM (7, 38). An et al. reported that blocking pyroptosis and NLRP3 inflammasome activation was effective in mitigating DM-related complications (39). Given that NLRP3 inflammasome was considered as a critical inducer for pyroptosis, we further explored whether miR-17-5p could inhibit pyroptosis in DM. Our result showed that cleavage of GSDMD was increased significantly in the pancreas of DM mice, indicating the presence of pyroptosis. By up-regulating the expression of miR-17-5p in DM mice, we found that DM-induced cleavage of GSDMD was inhibited. To confirm the anti-pyroptotic effect of miR-17-5p on INS-1 cells, flow cytometry was performed. Correspondingly, miR-17-5p mimic significantly reduced the population of pyroptotic cells induced by high glucose in INS-1 cells. Taken together, our data demonstrated that TXNIP/NLRP3 inflammasome pathway-related pyroptosis was involved in the beneficial effect of miR-17-5p to protect pancreatic β-cell in DM.

## Conclusions

Overall, we demonstrated that overexpression of miR-17-5p ameliorates DM-induced aberrant metabolisms of insulin and blood glucose. Our study highlighted the importance of the protective effect of miR-17-5p by inhibiting the TXNIP/NLRP3 inflammasome pathway and pyroptosis, providing a more complete understanding of the therapeutical value of miR-17-5p in DM.

## Data Availability Statement

The original contributions presented in the study are included in the article/Supplementary Material, further inquiries can be directed to the corresponding authors.

## Ethics Statement

The animal study was reviewed and approved by Ethic Committees of Sun Yat-sen University.

## Author Contributions

HT obtained funding for the study. HT and YC designed the study and helped to revise the manuscript. GT established the animal model. GT, SL, JG, FD, and CZ performed the experiment work, results collection, and interpretation. SL drafted the first manuscript. All authors reviewed the study and approved the final version.

## Funding

This work was supported by the National Natural Science Foundation of China (Grant Nos. 81873514, 82170357, and 81570394) and Guangdong Natural Science Foundation (Grant Nos. 2021A1515011766 and 2017A030311017).

## Conflict of Interest

The authors declare that the research was conducted in the absence of any commercial or financial relationships that could be construed as a potential conflict of interest.

## Publisher's Note

All claims expressed in this article are solely those of the authors and do not necessarily represent those of their affiliated organizations, or those of the publisher, the editors and the reviewers. Any product that may be evaluated in this article, or claim that may be made by its manufacturer, is not guaranteed or endorsed by the publisher.

## Abbreviations

DM: diabetes mellitus
NLRP3: Nod-like receptor protein 3
GSDMD: gasdermin D
GSDMD-NT: N-terminal GSDMD fragment
TXNIP: thioredoxin-interacting protein
mRNA: messenger RNAs
STZ: streptozotocin
AUC: area under the curve
qRT-PCR: quantitative real-time polymerase chain reaction
7-AAD: 7-aminoactinomycin D.
