# Supplementary figures and images for: MiR-17-5p Inhibits TXNIP/NLRP3 Inflammasome Pathway and Suppresses Pancreatic β-Cell Pyroptosis in Diabetic Mice
Source: Front Cardiovasc Med. 2021 Nov 22;8:768029. doi: 10.3389/fcvm.2021.768029 (PMC8645844; doi:10.3389/fcvm.2021.768029)

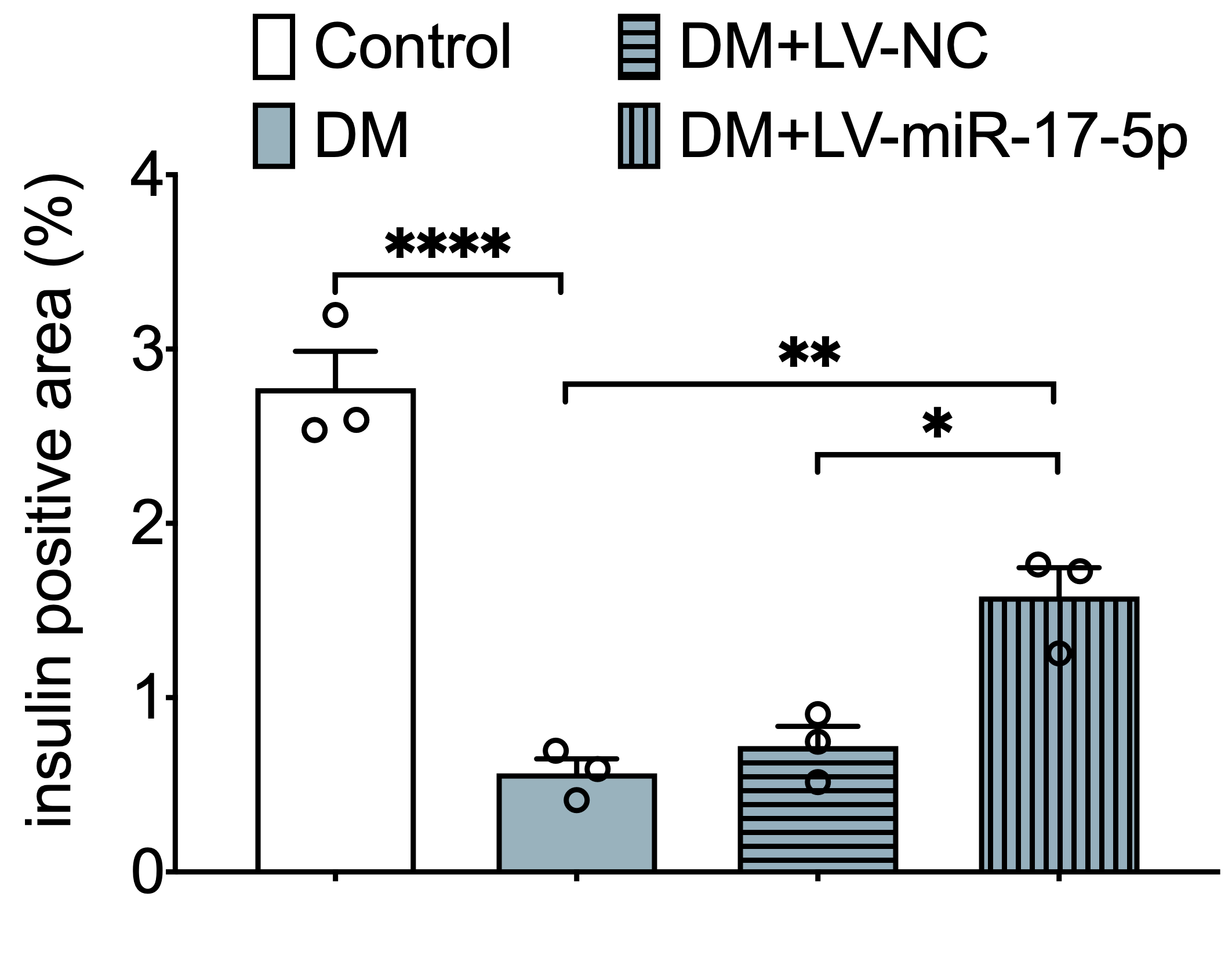

Supplement: Supplementary file 1 [file Data_Sheet_1.ZIP › figure E-F/statistics/11insulin positive area.tiff]

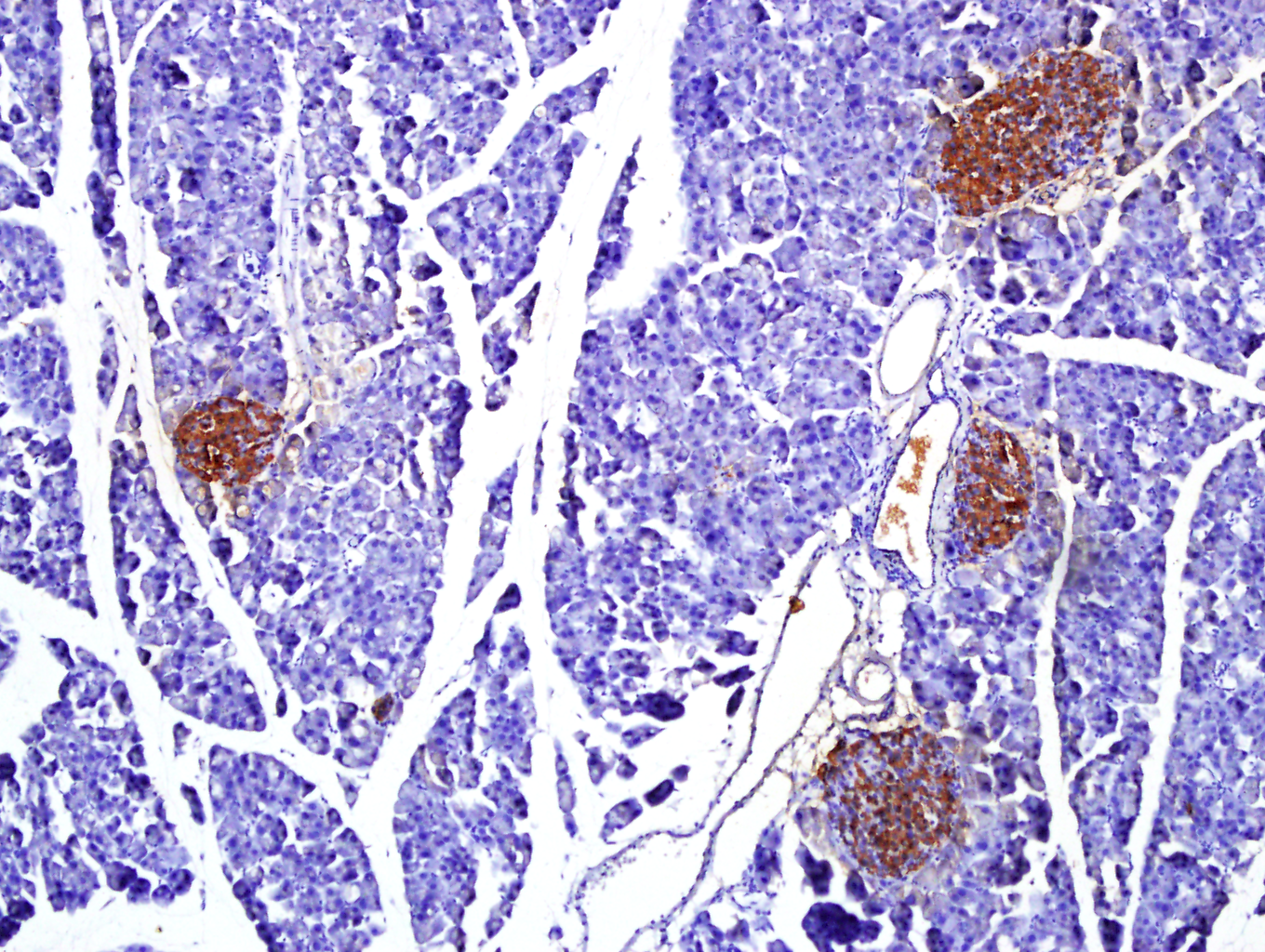

Supplement: Supplementary file 1 [file Data_Sheet_1.ZIP › figure E-F/picture/CT1-4-10x.tif]

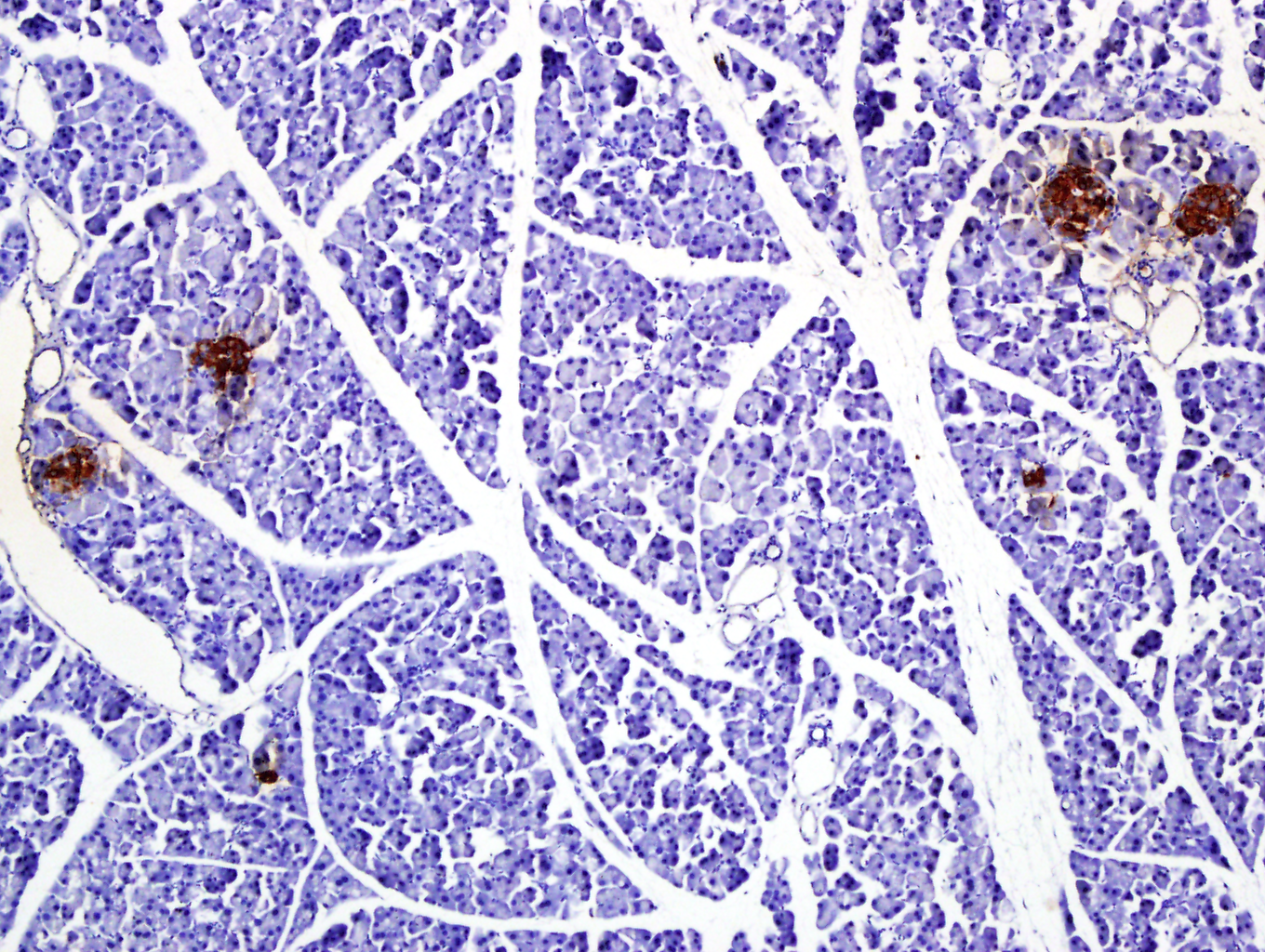

Supplement: Supplementary file 1 [file Data_Sheet_1.ZIP › figure E-F/picture/NC1-4-10x.tif]

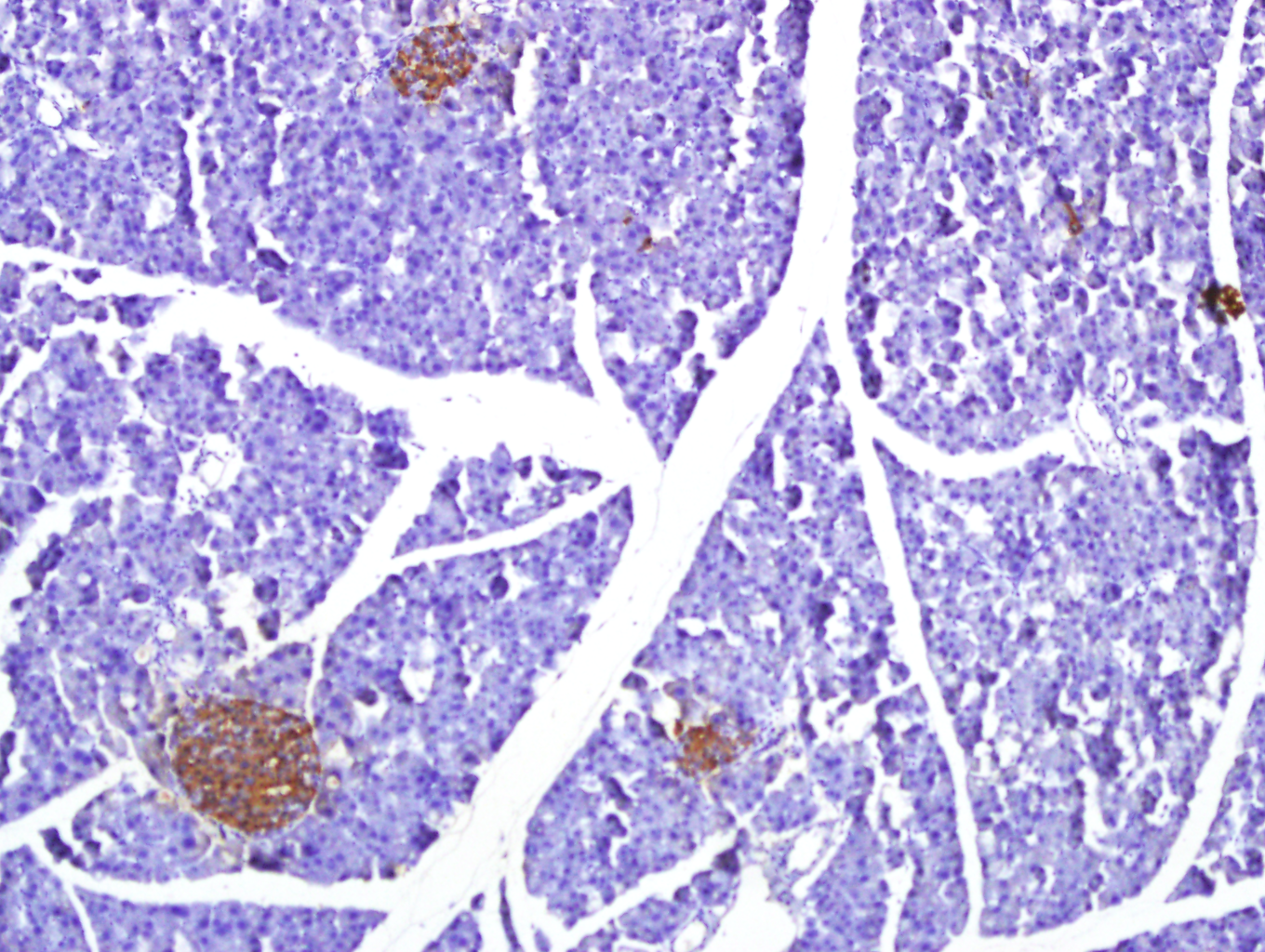

Supplement: Supplementary file 1 [file Data_Sheet_1.ZIP › figure E-F/picture/MIMIC1-2-10x.tif]

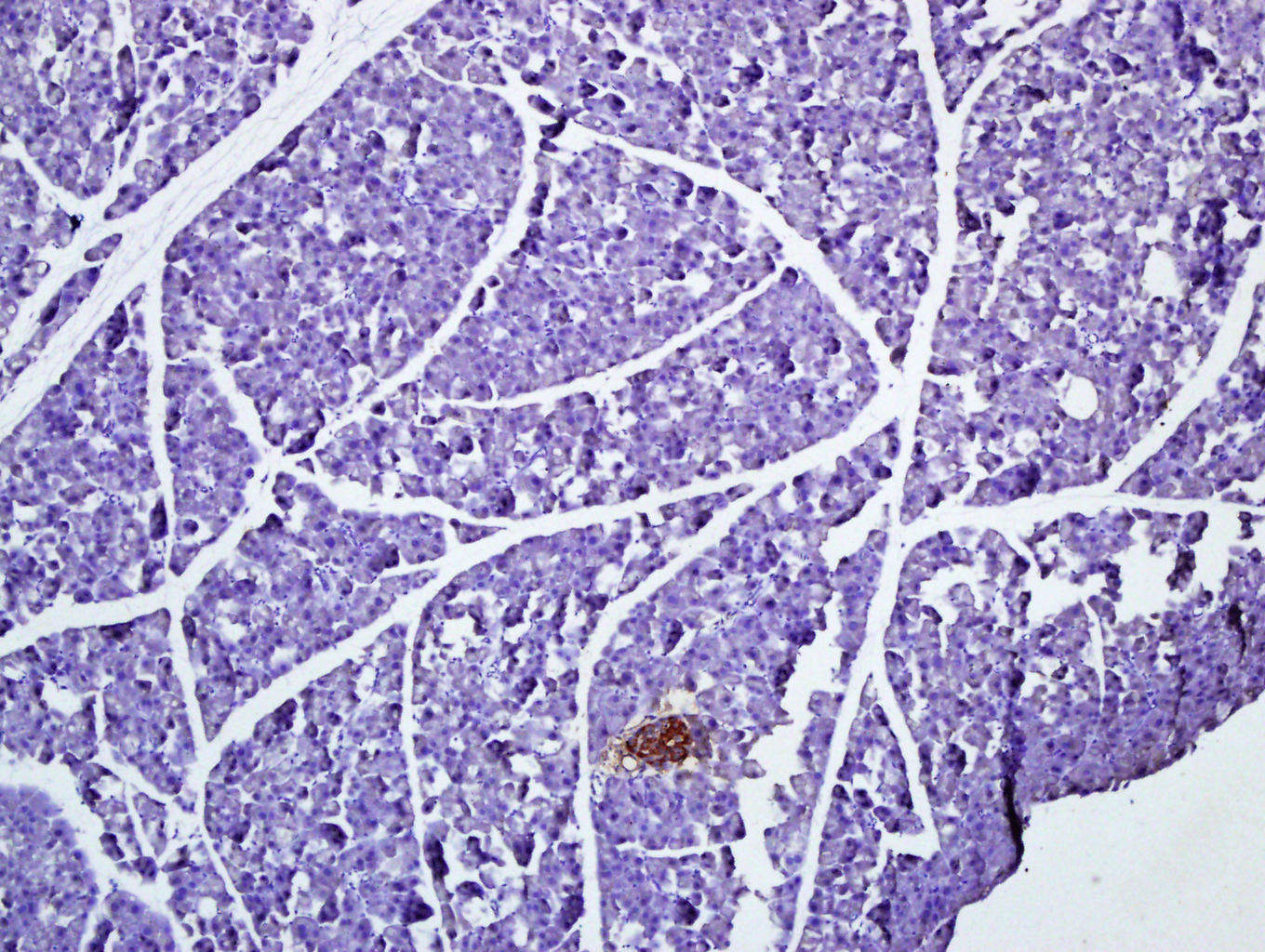

Supplement: Supplementary file 1 [file Data_Sheet_1.ZIP › figure E-F/picture/DM1-4-10x.tif]

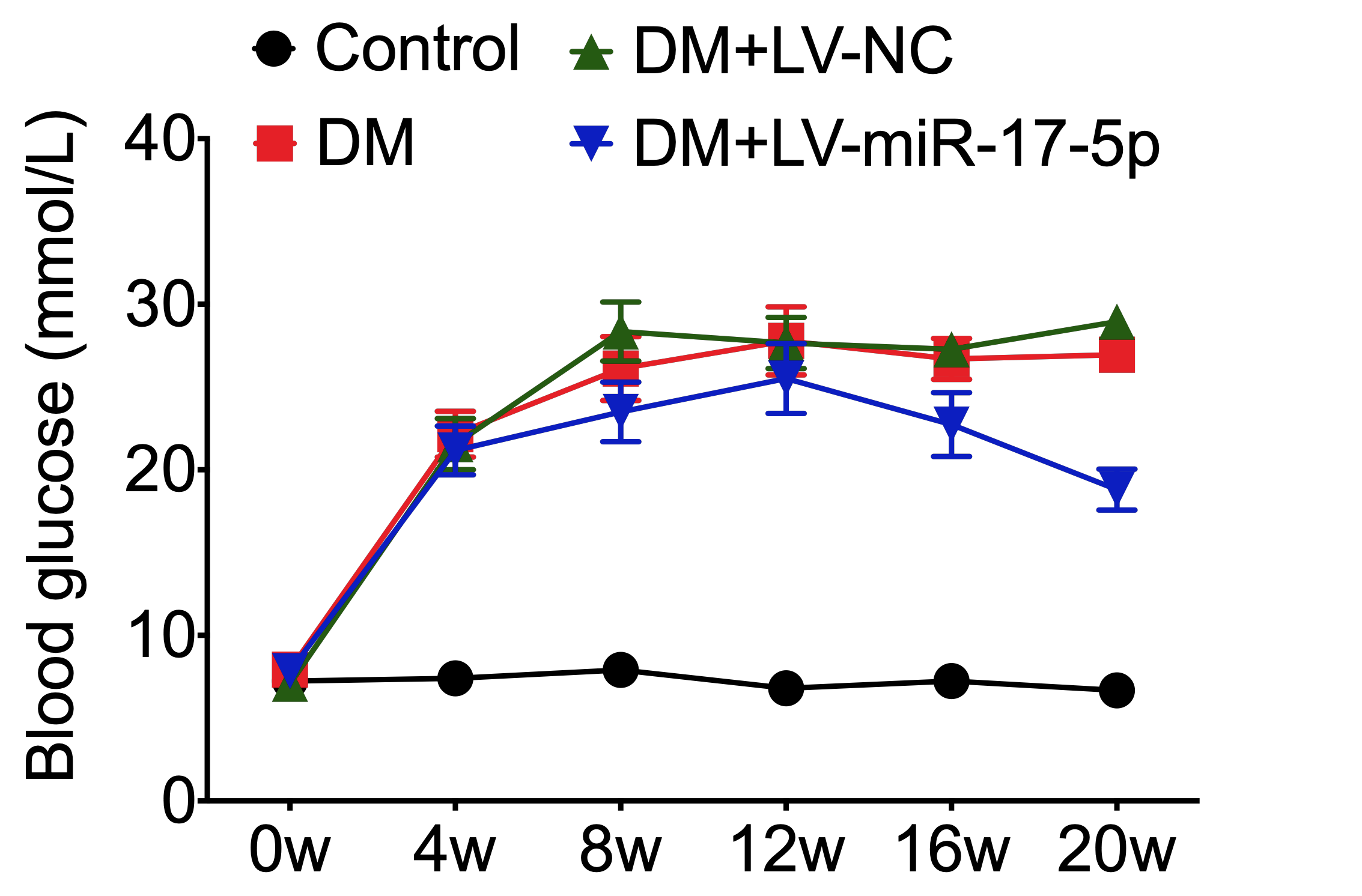

Supplement: Supplementary file 2 [file Data_Sheet_2.ZIP › data8.30/figure4/figureB/blood glucose.tiff]

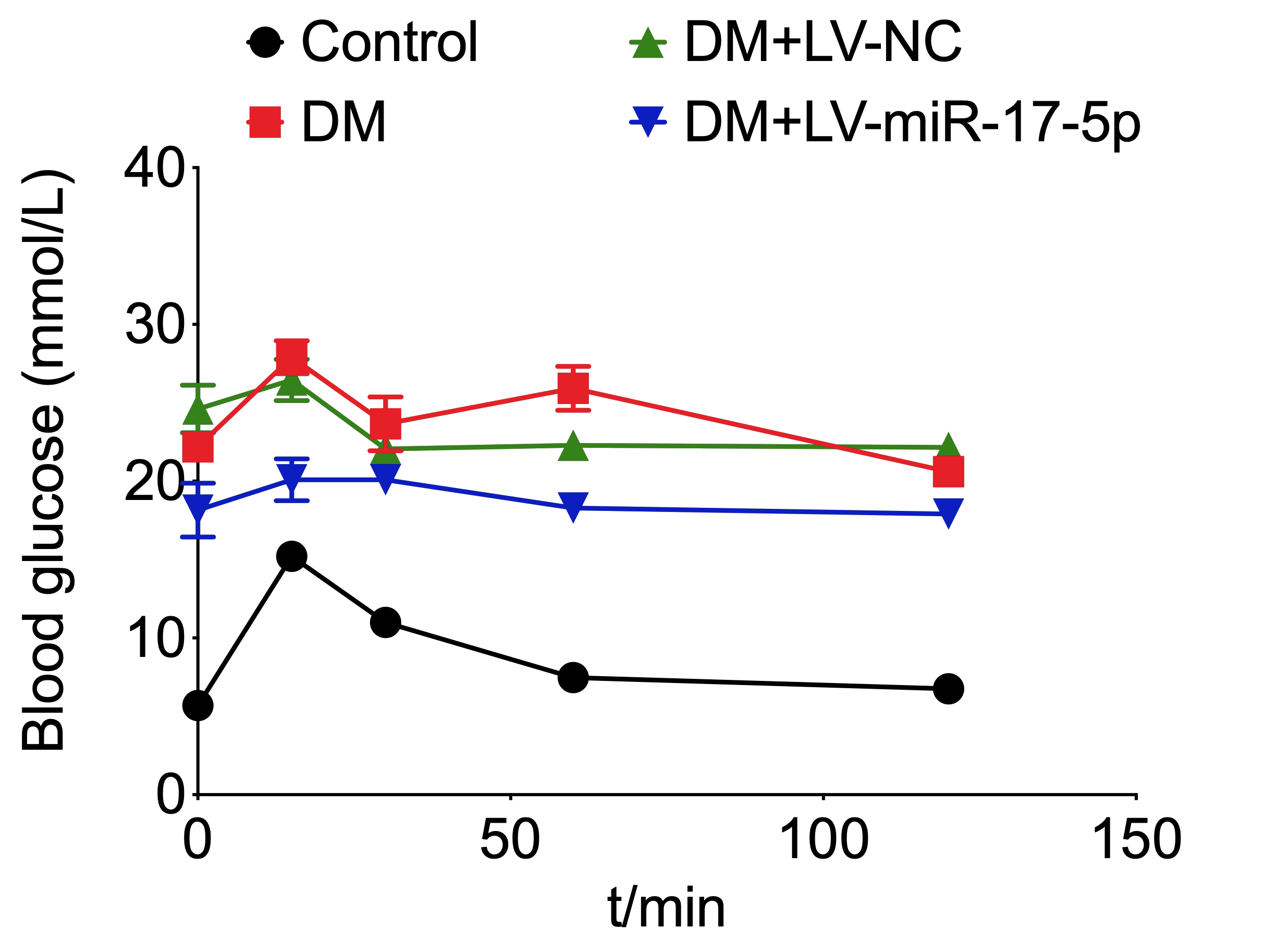

Supplement: Supplementary file 2 [file Data_Sheet_2.ZIP › data8.30/figure4/figure C-D/figure4c.tiff]

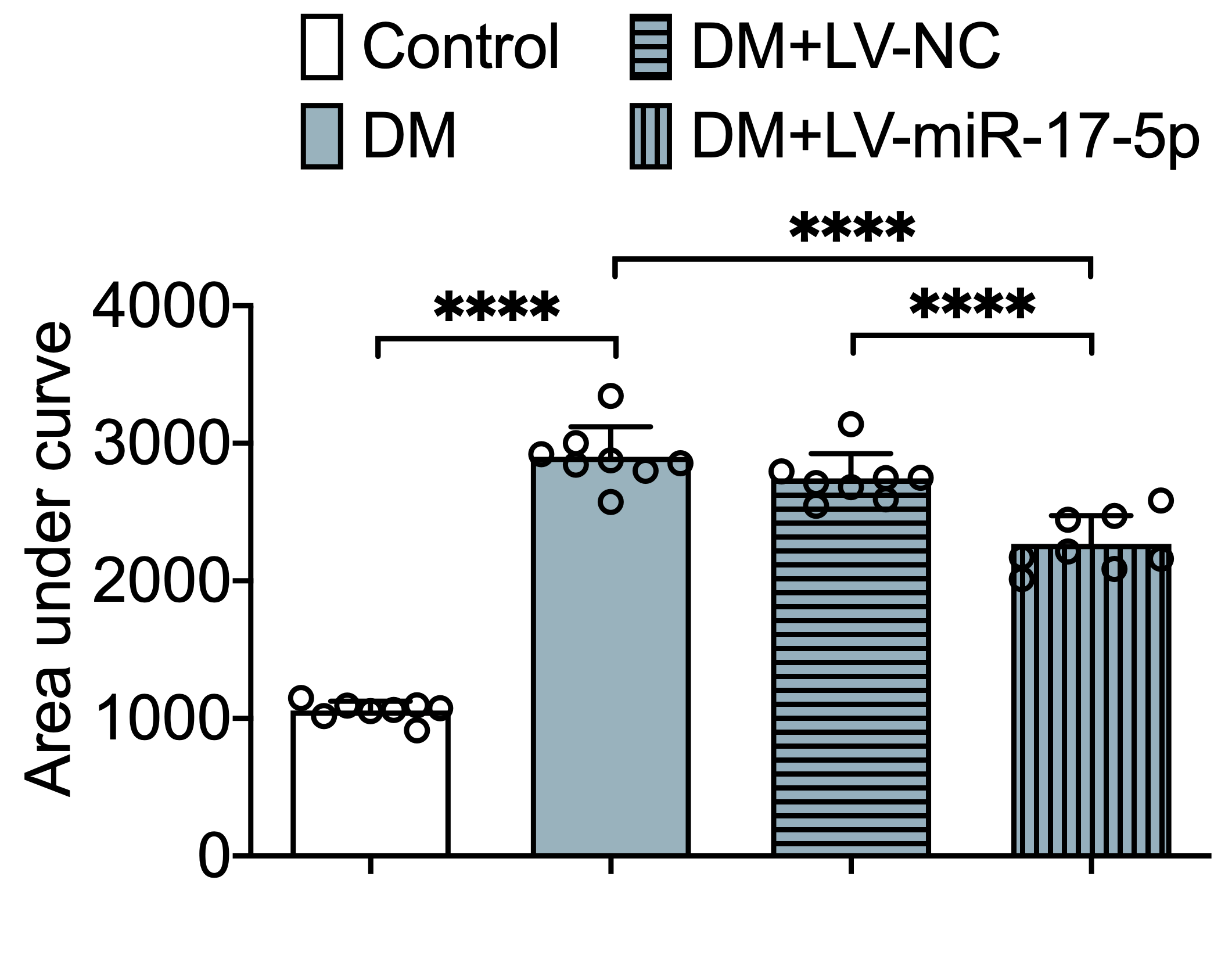

Supplement: Supplementary file 2 [file Data_Sheet_2.ZIP › data8.30/figure4/figure C-D/figure4d.tiff]

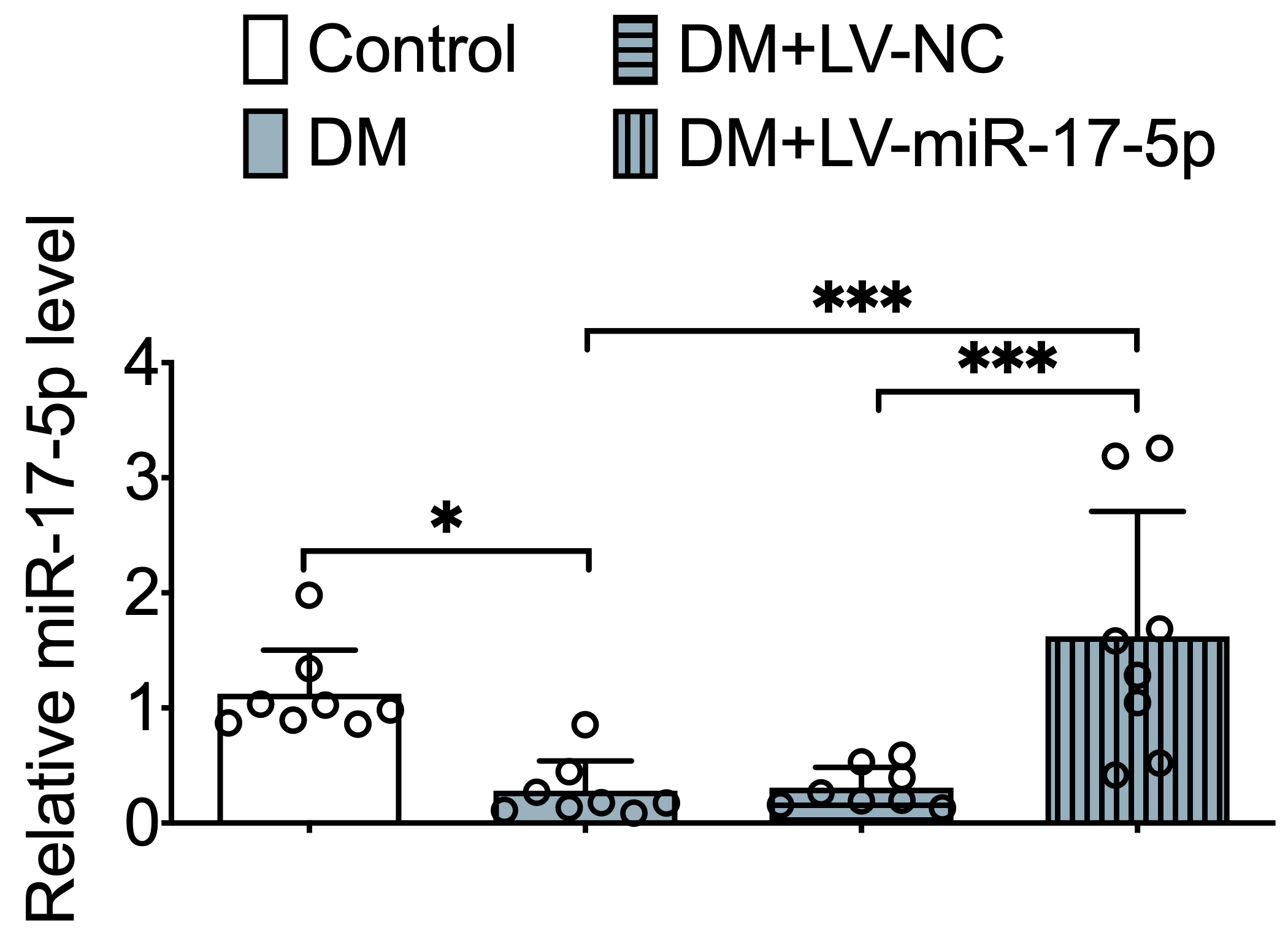

Supplement: Supplementary file 2 [file Data_Sheet_2.ZIP › data8.30/figure4/figure4a/figure4a-.tiff]

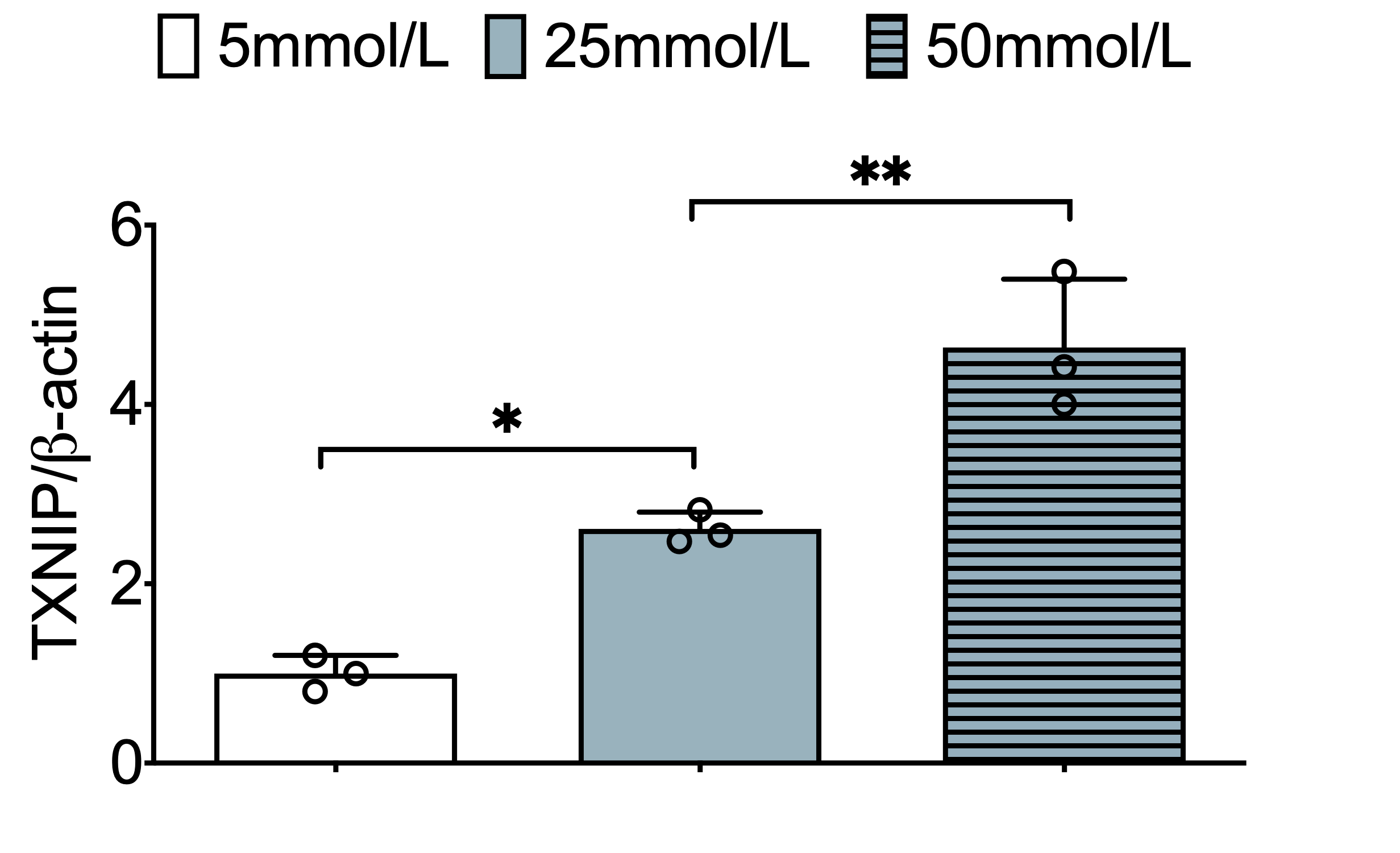

Supplement: Supplementary file 2 [file Data_Sheet_2.ZIP › data8.30/figure1/figure 1CD/statistics/HGσ╜▒σôì -TXNIP.tiff]

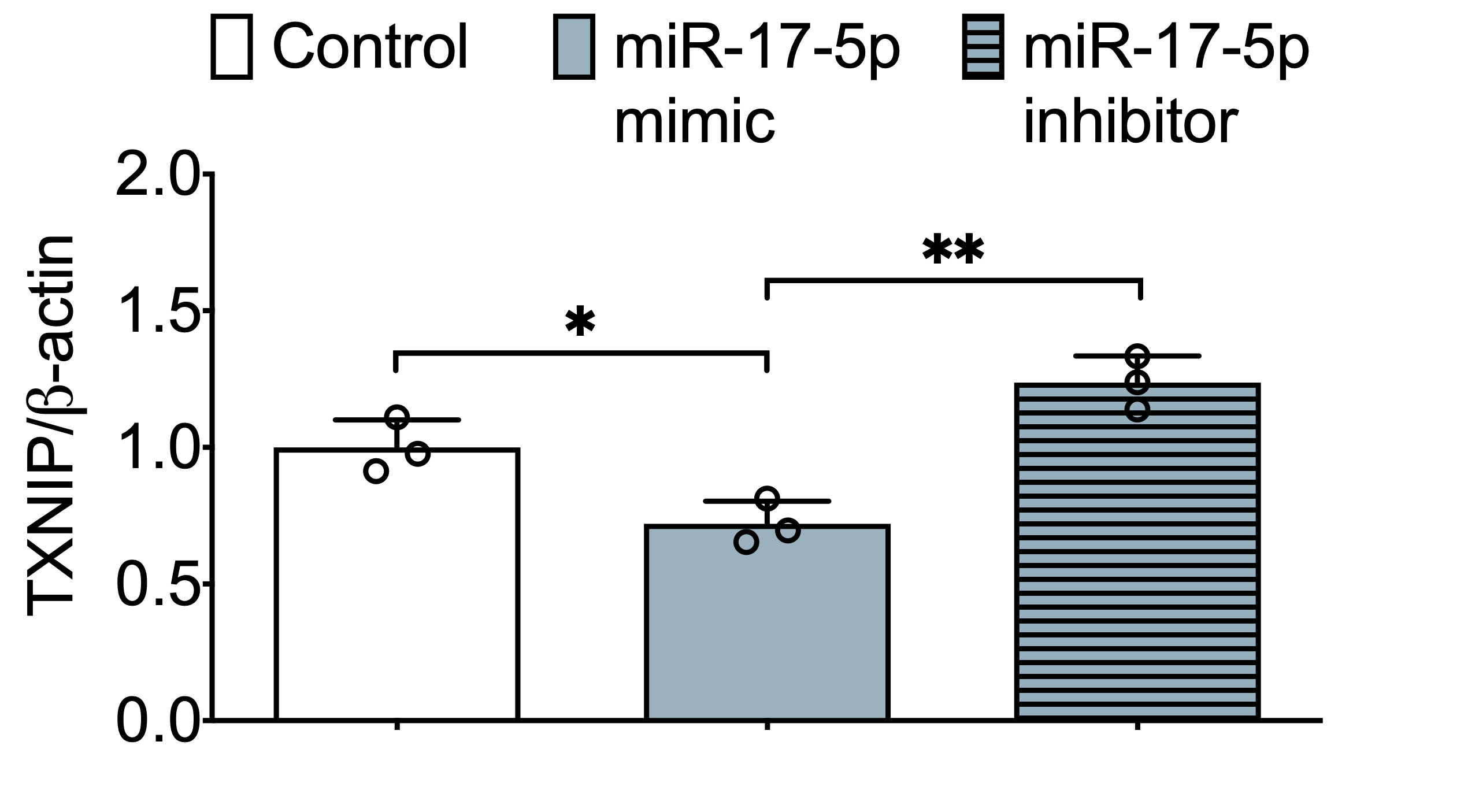

Supplement: Supplementary file 2 [file Data_Sheet_2.ZIP › data8.30/figure1/figure 1CD/statistics/inhibitor.tiff]

Glucose mmol/L

48h                      72h  
5    25    50        5    25    50

48h                      72h  
5    25    50        5    25    50

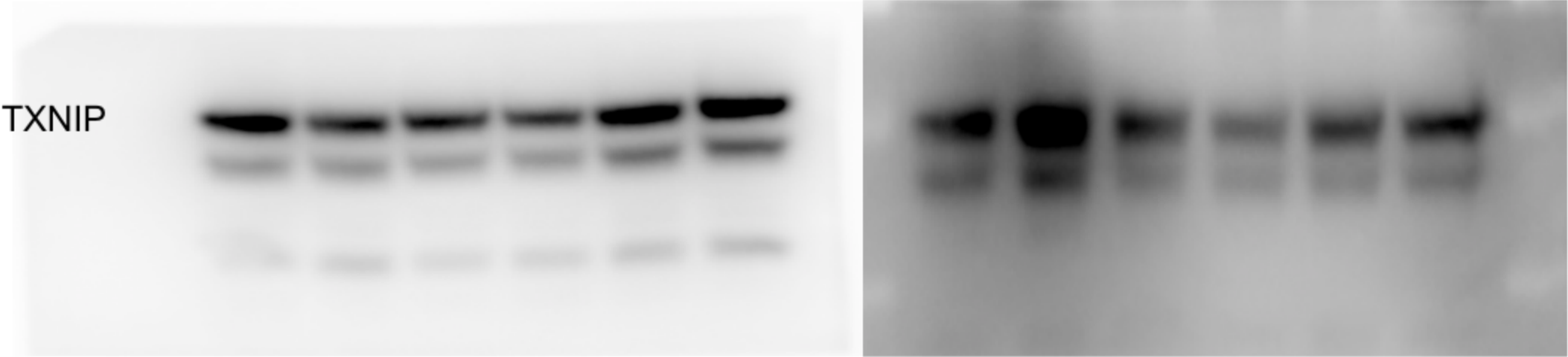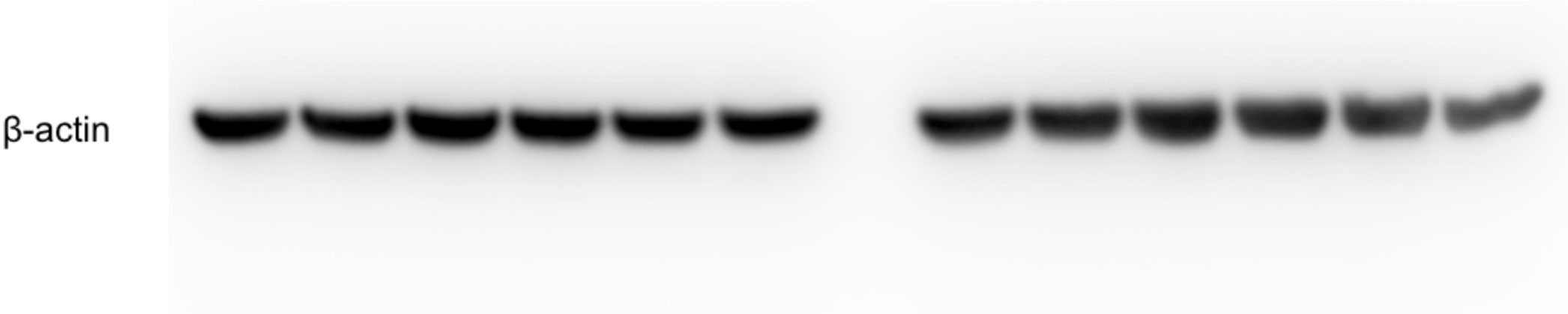

Glucose mmol/L

72h

5

25

50

Txnip

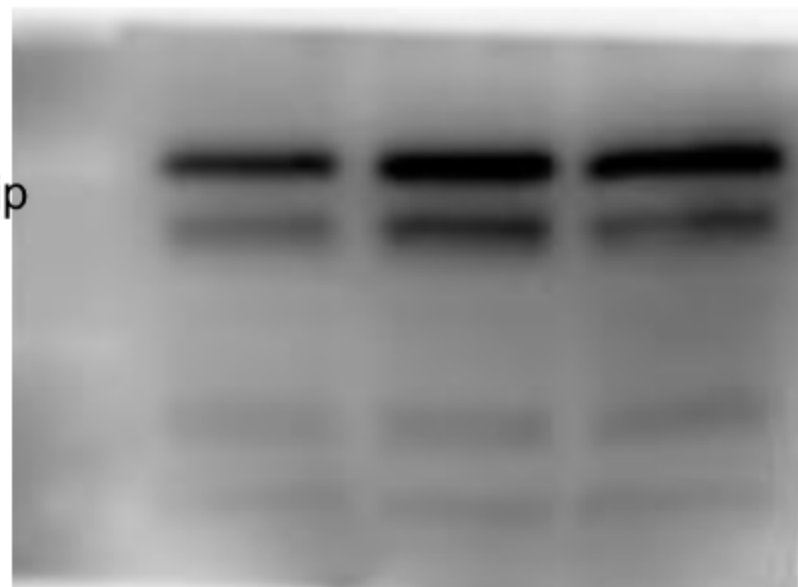

$\beta$ -actin

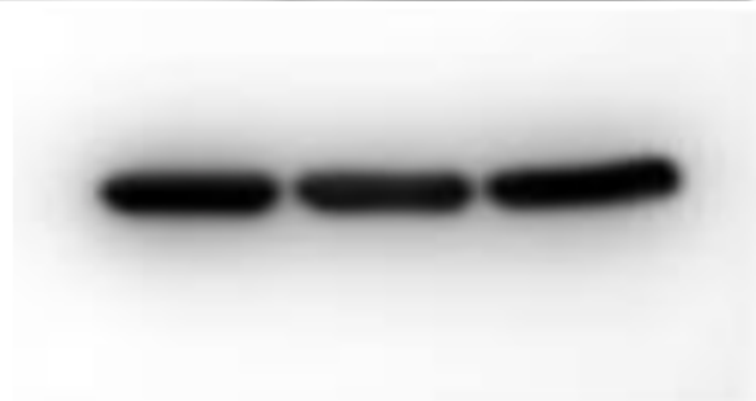

Supplement: Supplementary file 2 [file Data_Sheet_2.ZIP › data8.30/figure1/figure 1CD/picture/figure 1B.pdf]

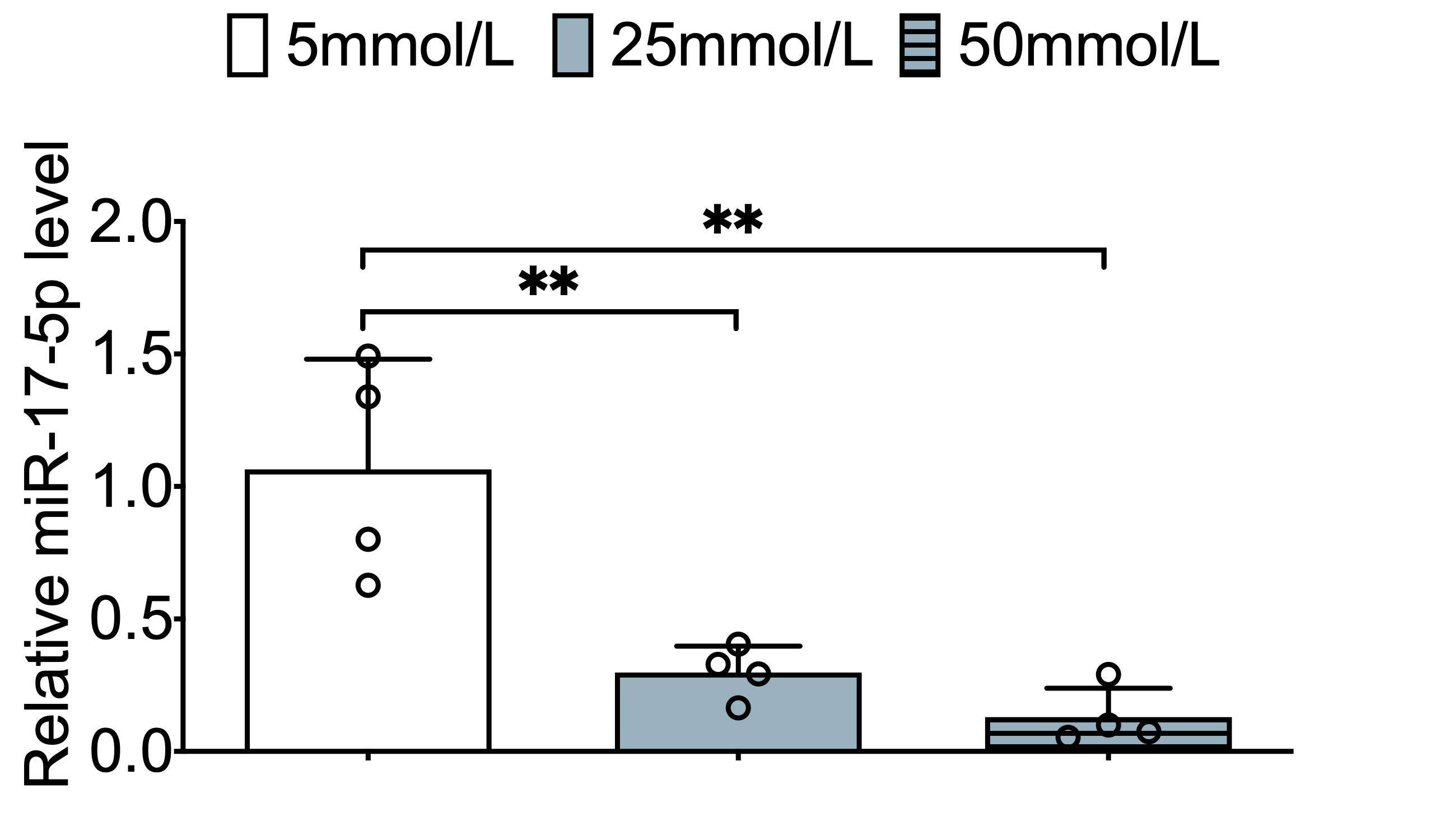

Supplement: Supplementary file 2 [file Data_Sheet_2.ZIP › data8.30/figure1/figure1a/statistics.tiff]

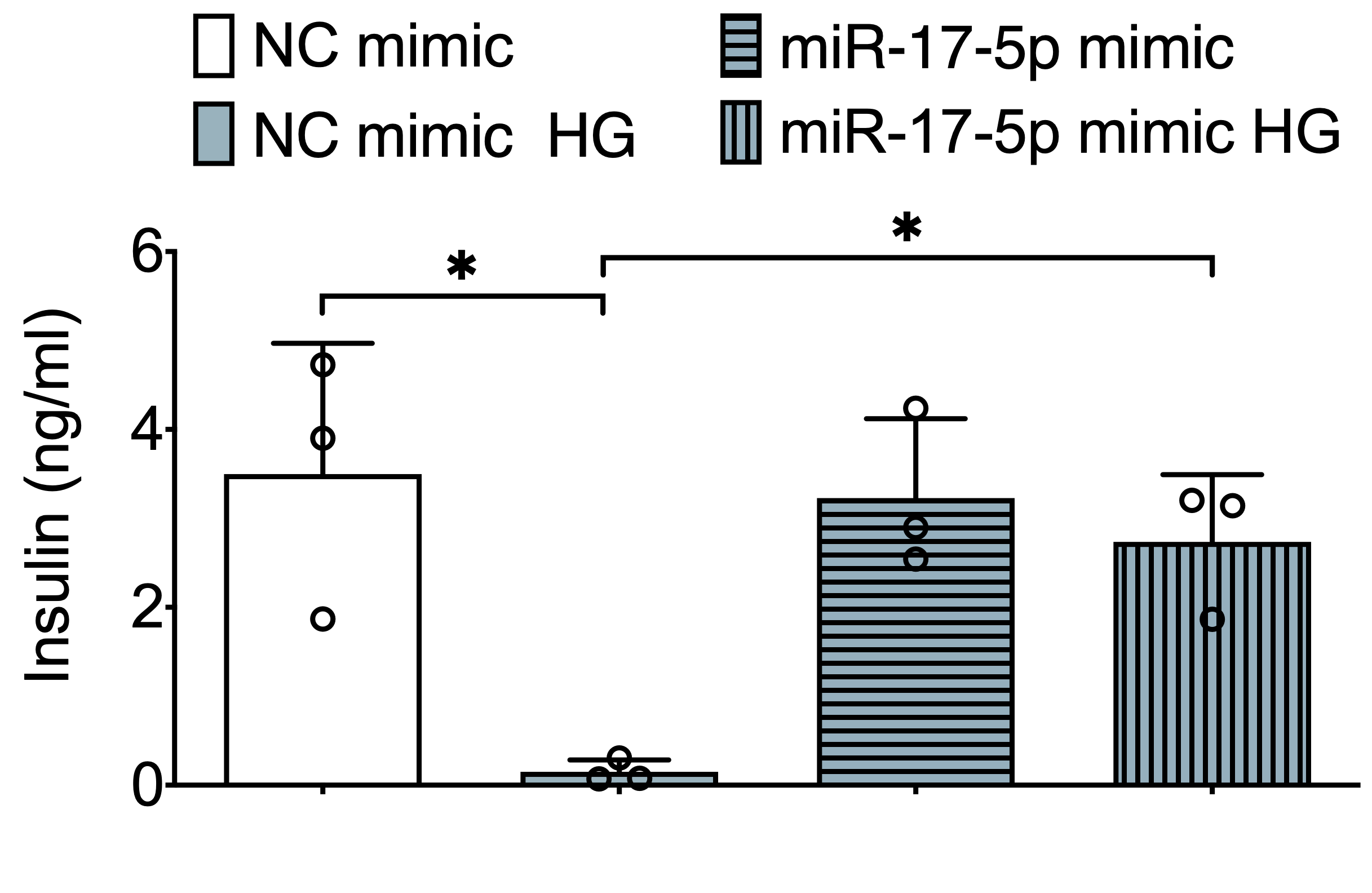

Supplement: Supplementary file 2 [file Data_Sheet_2.ZIP › data8.30/figure1/figure1B/insulin.tiff]

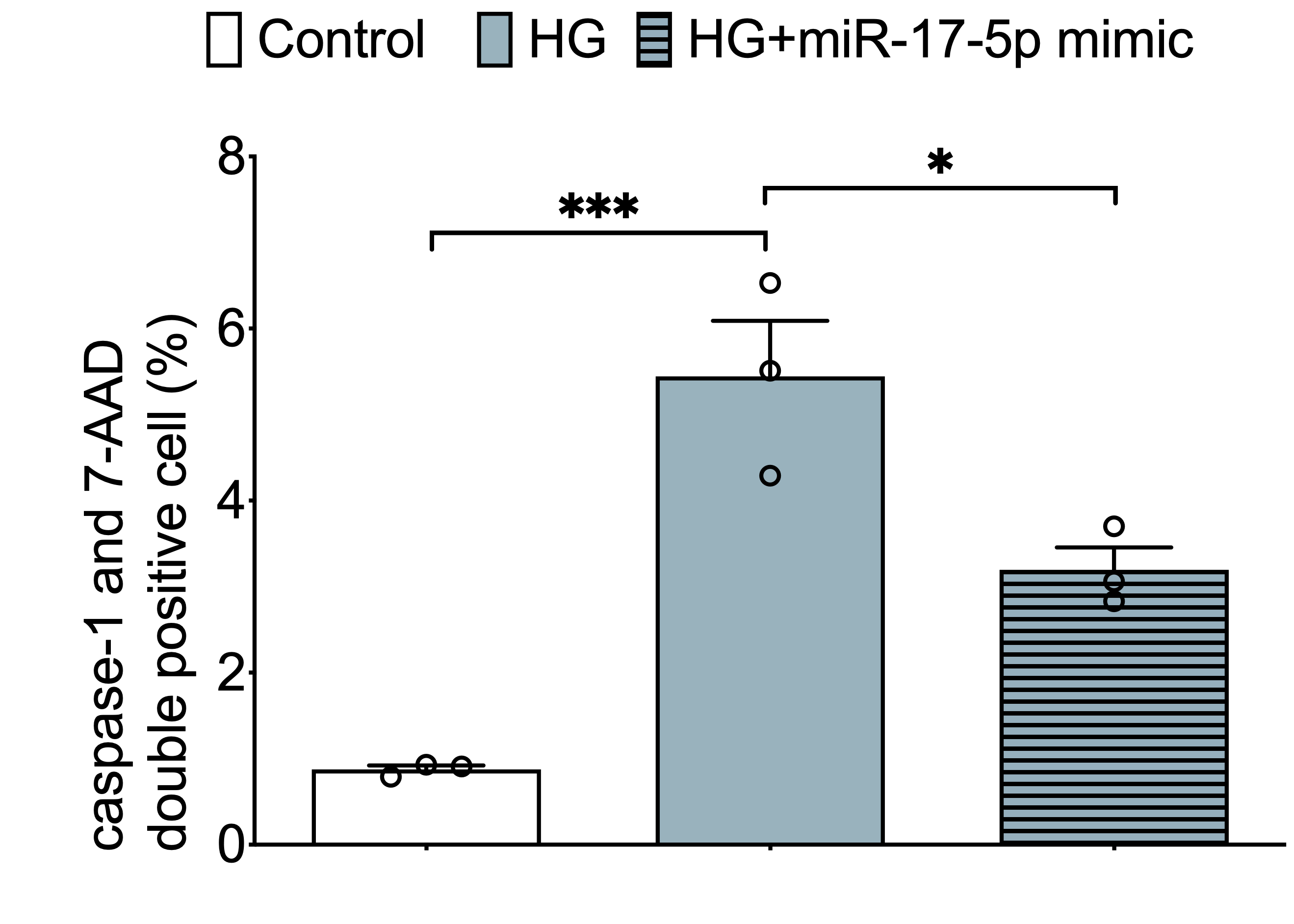

Supplement: Supplementary file 2 [file Data_Sheet_2.ZIP › data8.30/figure2-3/figure3cd/statistics/Data- 1.tiff]

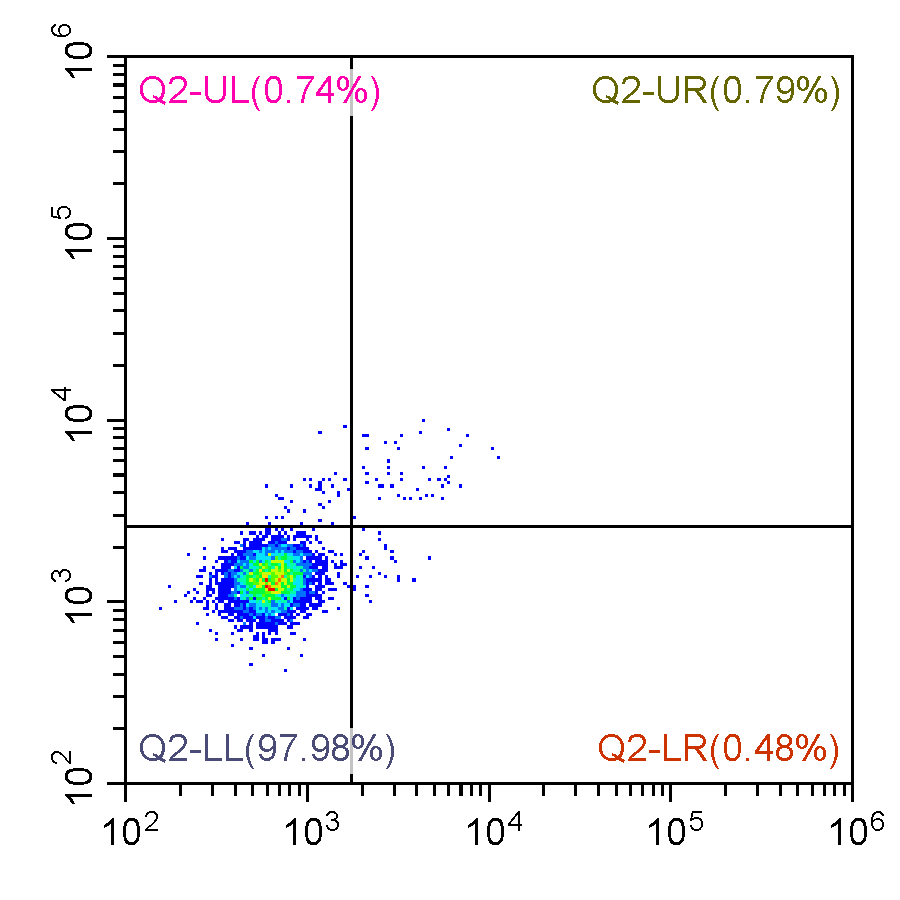

Supplement: Supplementary file 2 [file Data_Sheet_2.ZIP › data8.30/figure2-3/figure3cd/picture/control-2.bmp]

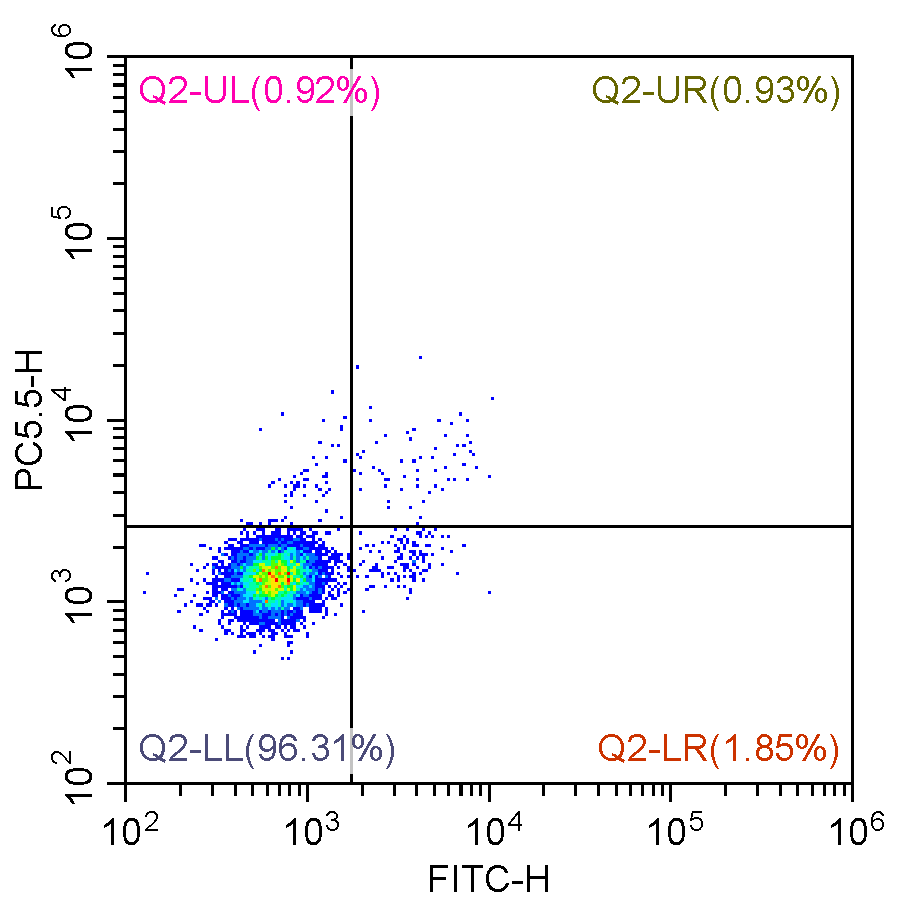

Supplement: Supplementary file 2 [file Data_Sheet_2.ZIP › data8.30/figure2-3/figure3cd/picture/control-3.bmp]

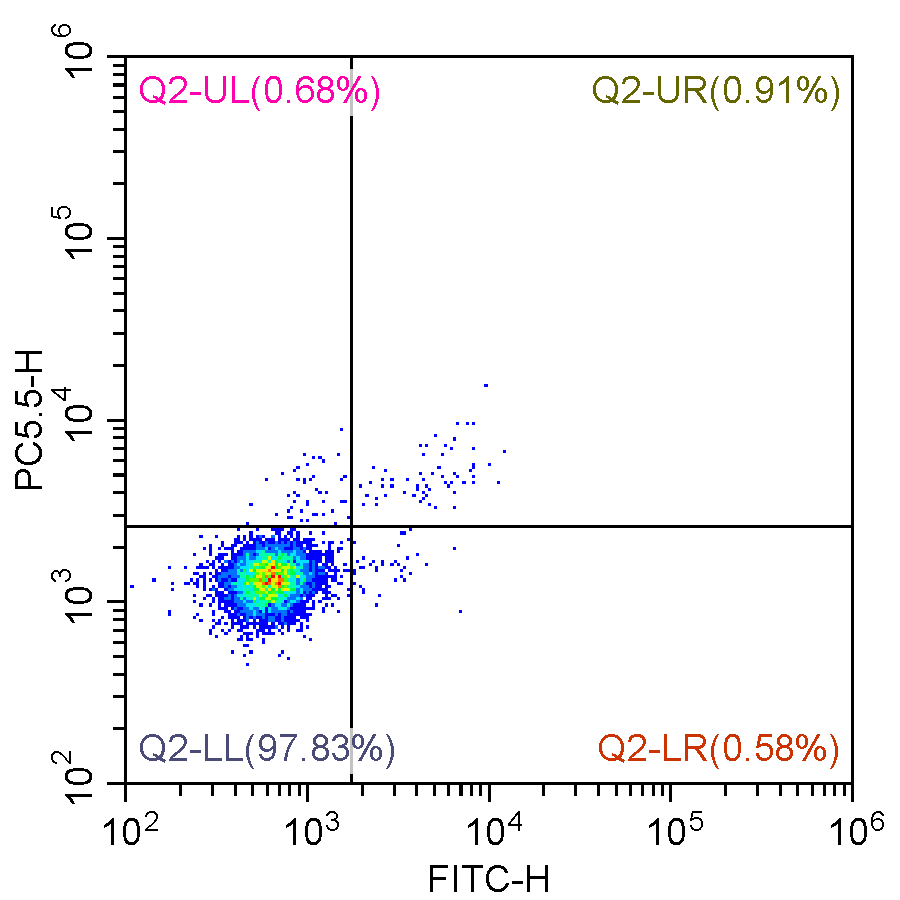

Supplement: Supplementary file 2 [file Data_Sheet_2.ZIP › data8.30/figure2-3/figure3cd/picture/control-1.bmp]

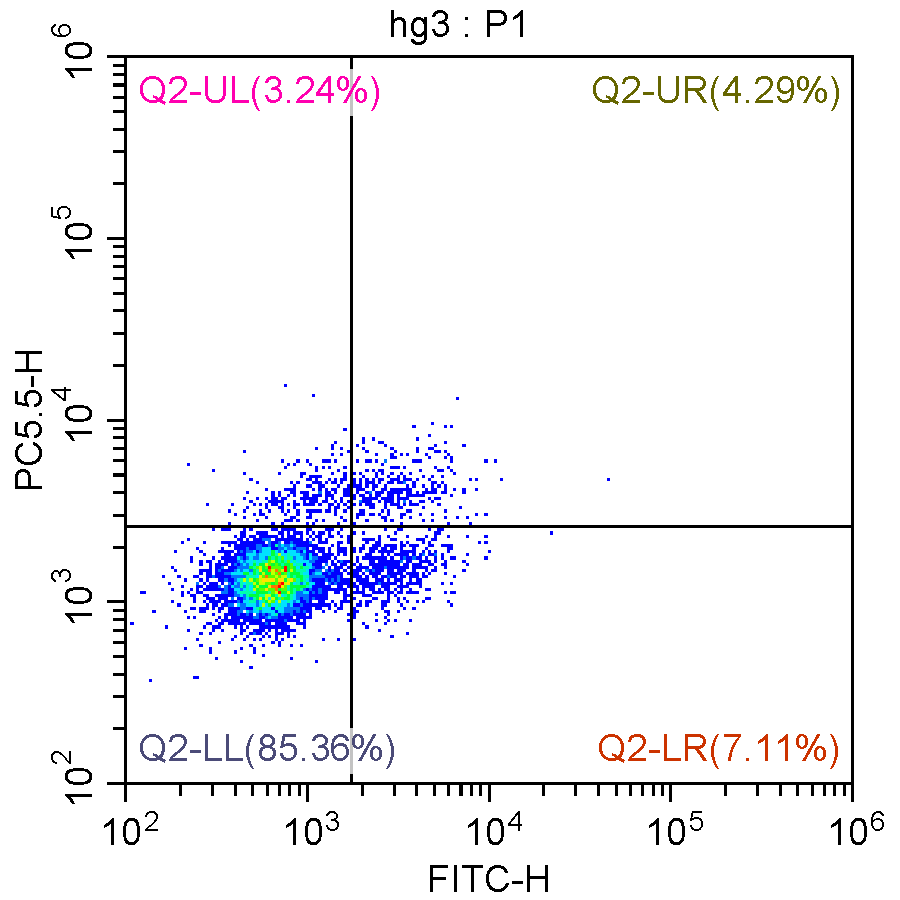

Supplement: Supplementary file 2 [file Data_Sheet_2.ZIP › data8.30/figure2-3/figure3cd/picture/HG3.bmp]

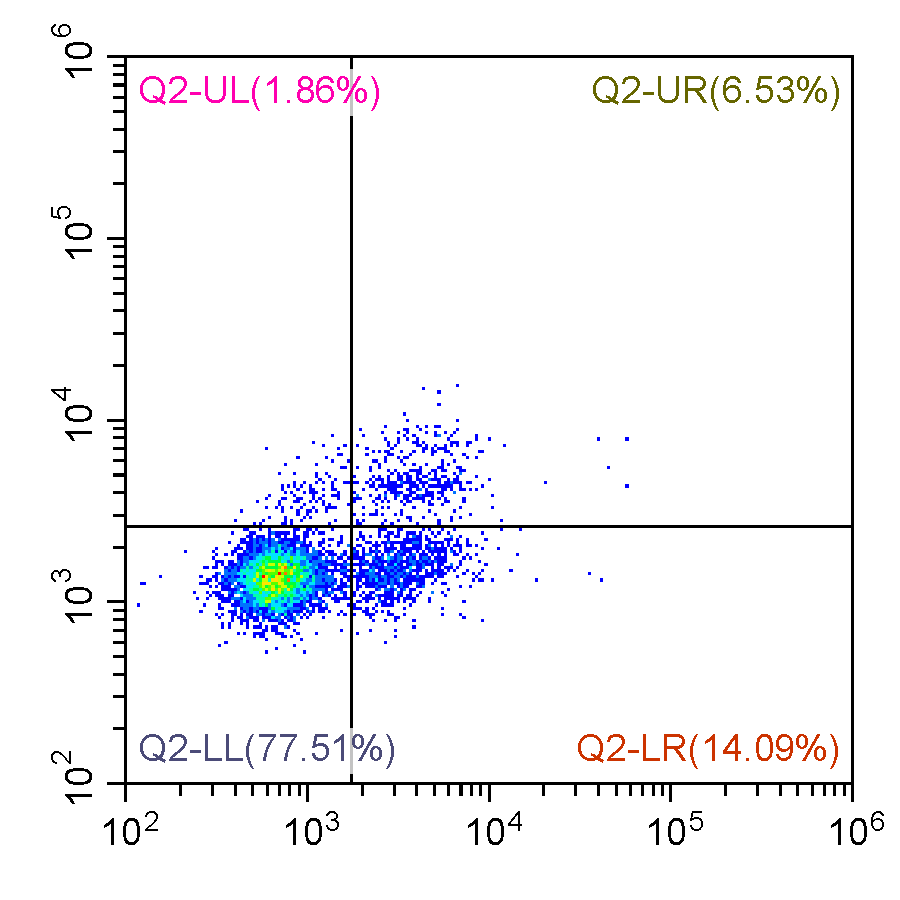

Supplement: Supplementary file 2 [file Data_Sheet_2.ZIP › data8.30/figure2-3/figure3cd/picture/HG2.bmp]

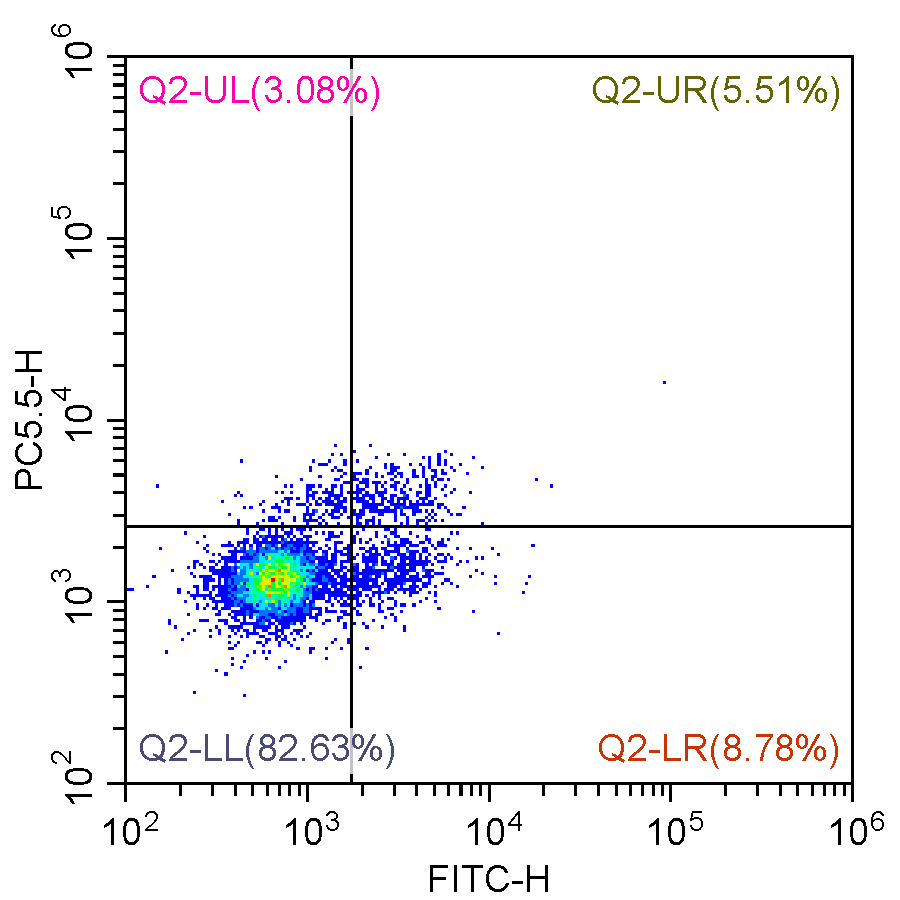

Supplement: Supplementary file 2 [file Data_Sheet_2.ZIP › data8.30/figure2-3/figure3cd/picture/HG1.bmp]

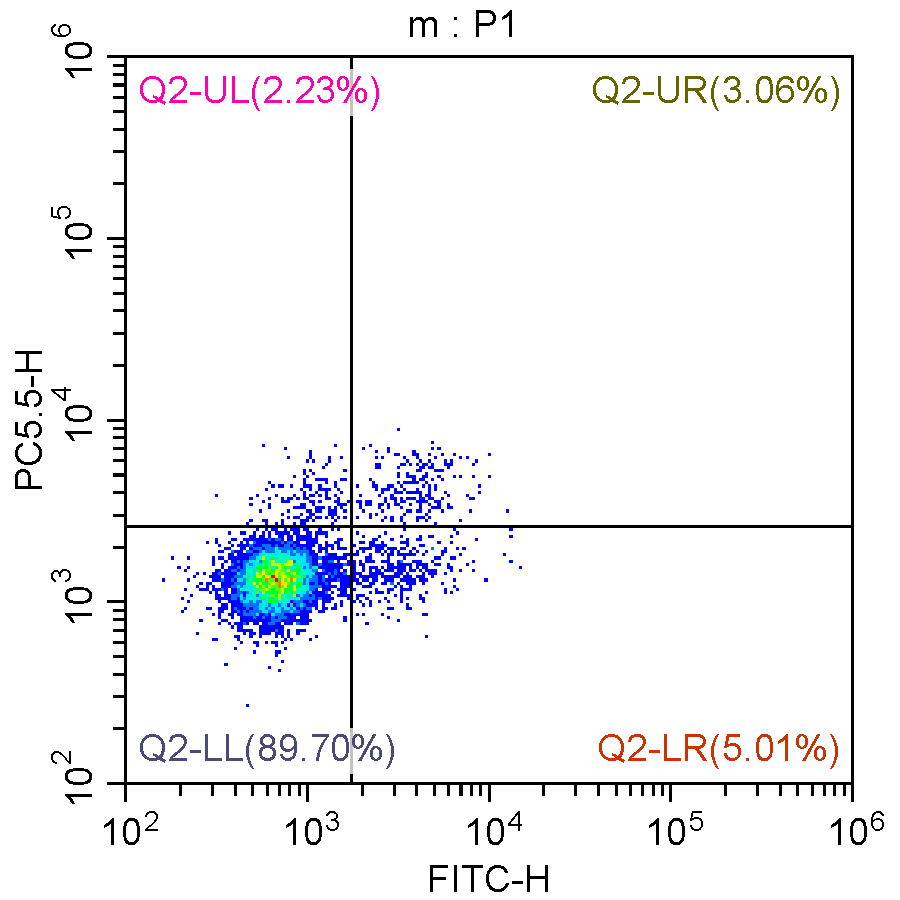

Supplement: Supplementary file 2 [file Data_Sheet_2.ZIP › data8.30/figure2-3/figure3cd/picture/MIMIC1.bmp]

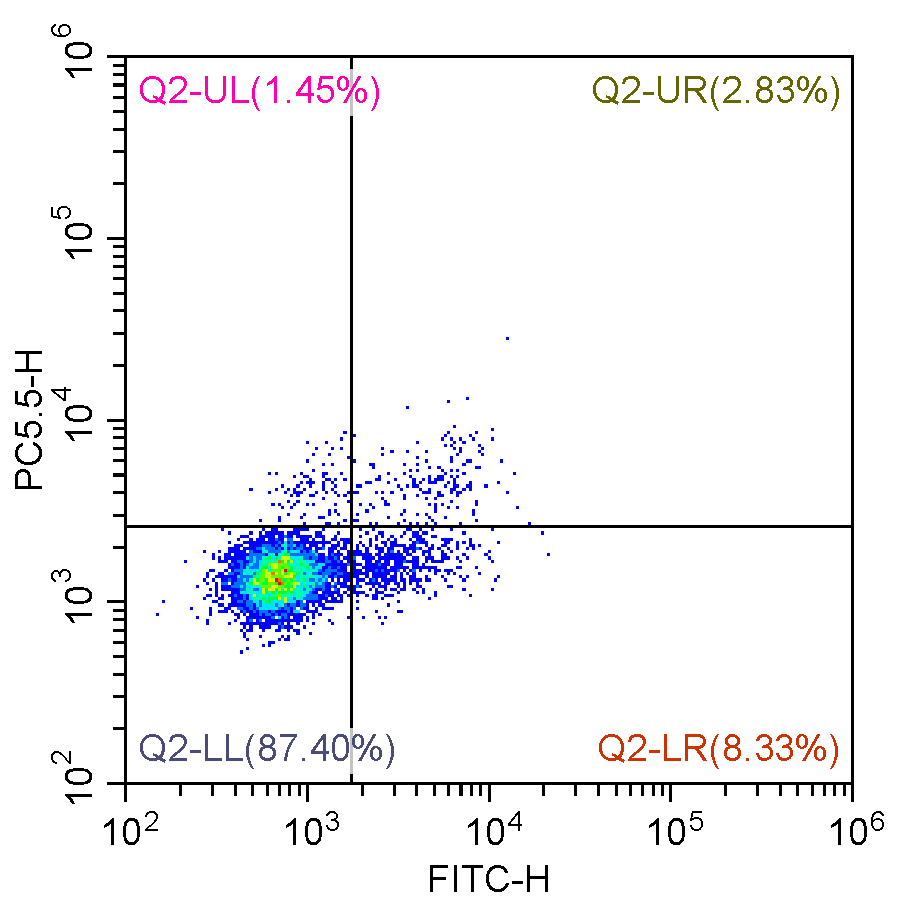

Supplement: Supplementary file 2 [file Data_Sheet_2.ZIP › data8.30/figure2-3/figure3cd/picture/MIMIC2.bmp]

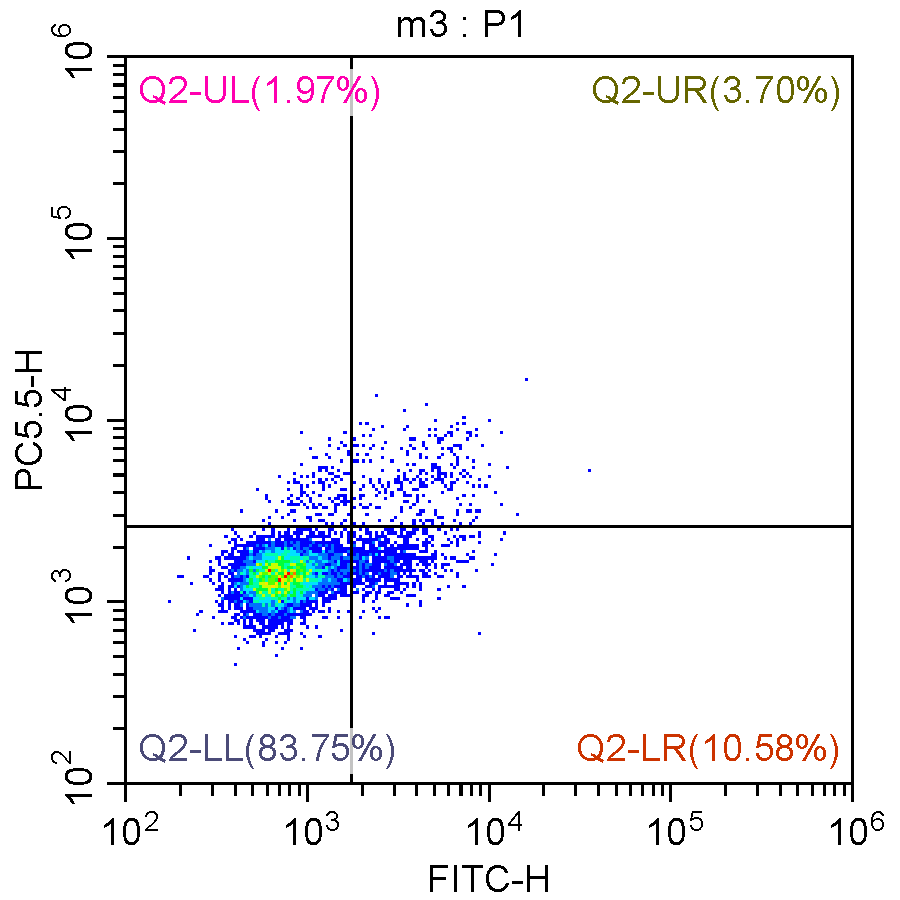

Supplement: Supplementary file 2 [file Data_Sheet_2.ZIP › data8.30/figure2-3/figure3cd/picture/MIMIC3.bmp]

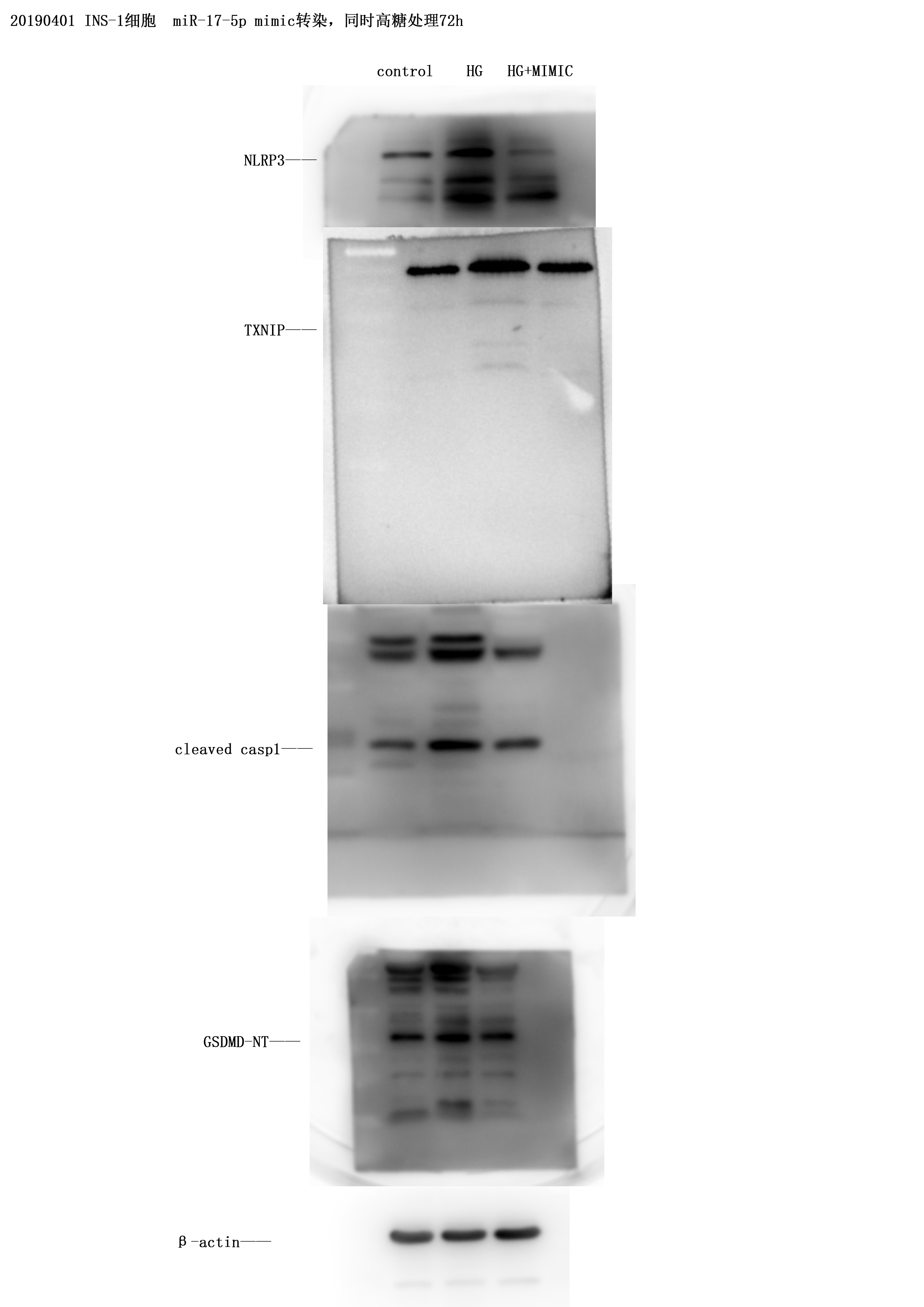

Supplement: Supplementary file 2 [file Data_Sheet_2.ZIP › data8.30/figure2-3/figure2ab-3abpicture/figure2a-1.tif]

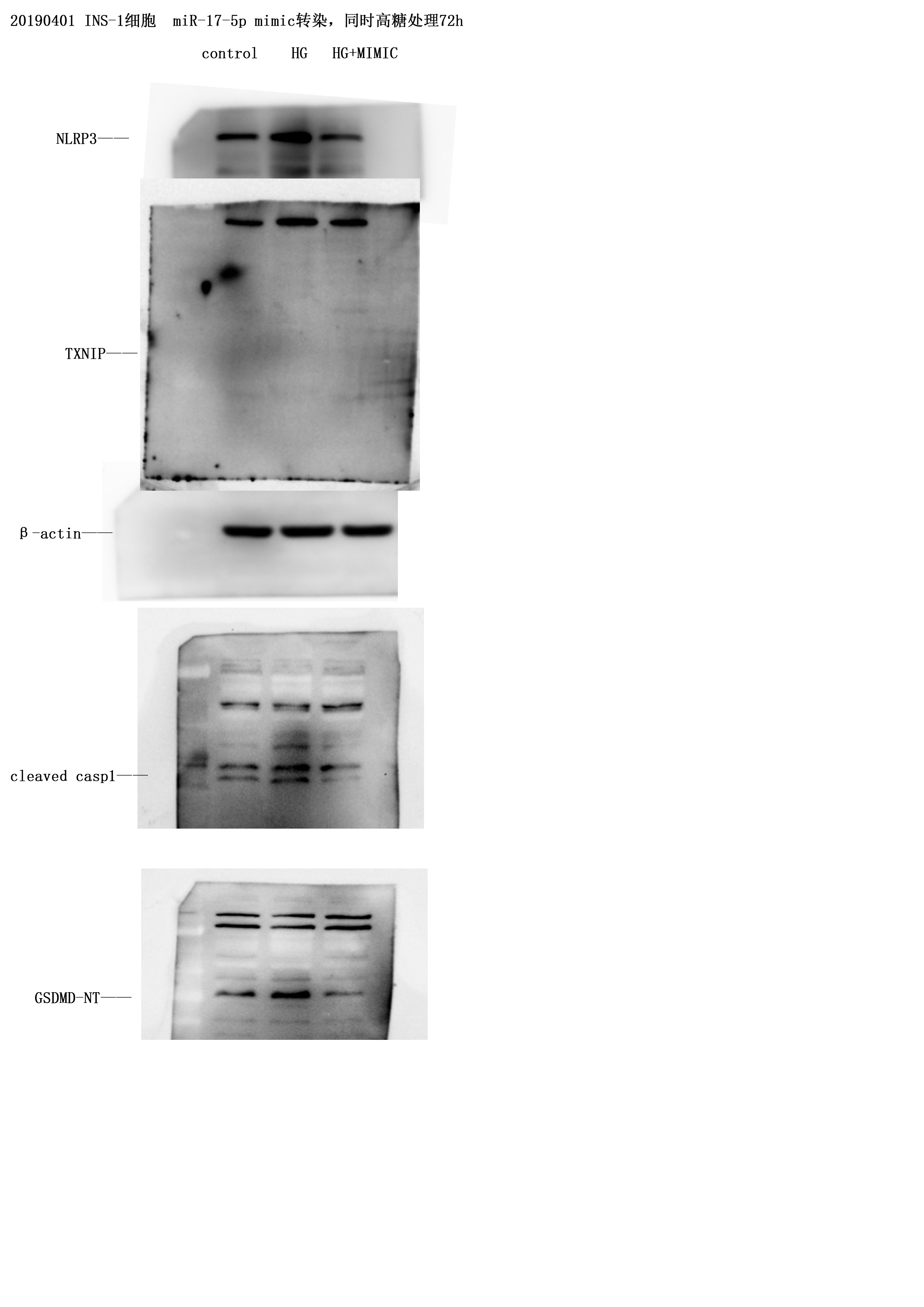

Supplement: Supplementary file 2 [file Data_Sheet_2.ZIP › data8.30/figure2-3/figure2ab-3abpicture/figure2a-3.tif]

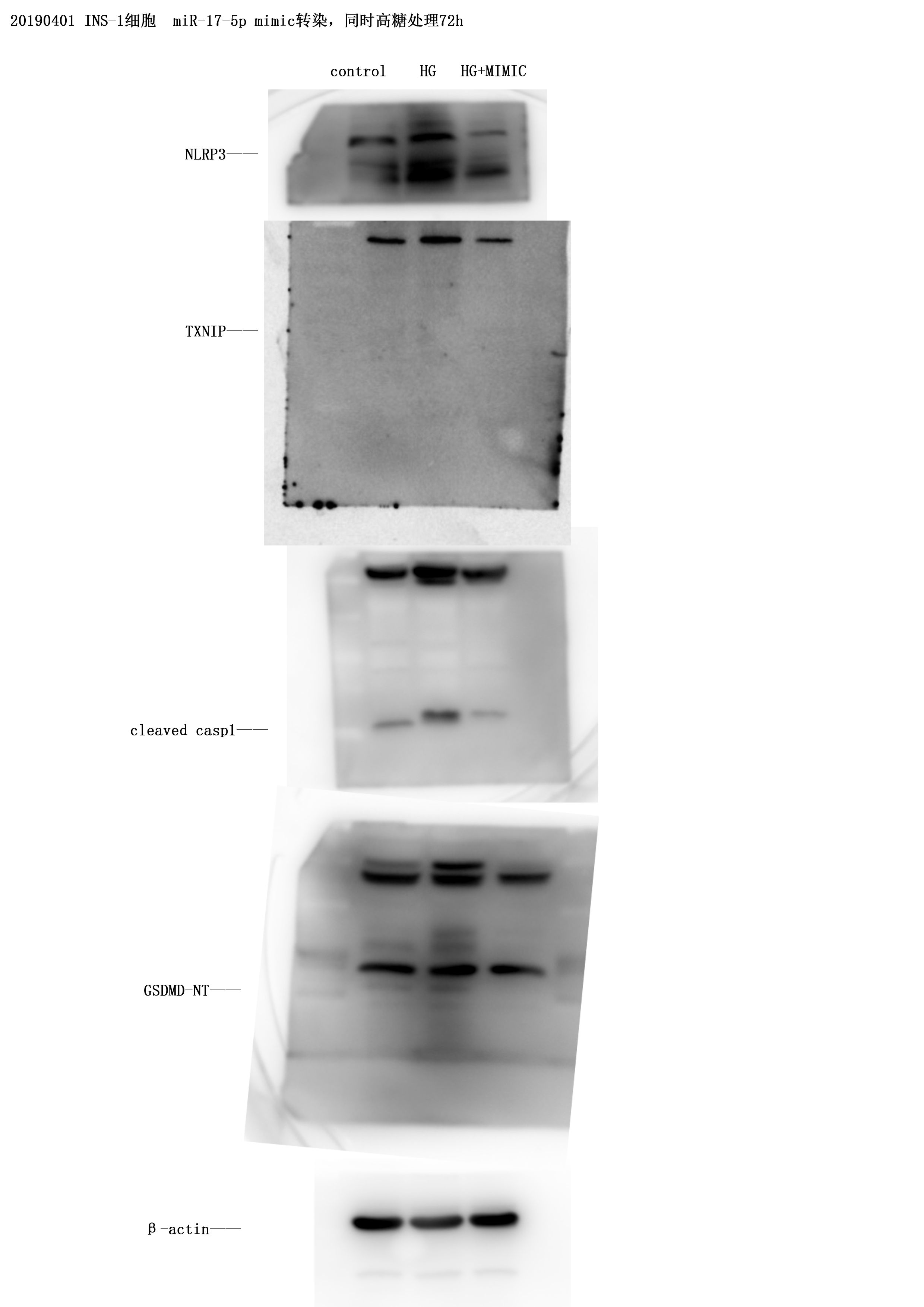

Supplement: Supplementary file 2 [file Data_Sheet_2.ZIP › data8.30/figure2-3/figure2ab-3abpicture/figure2a-2.tif]

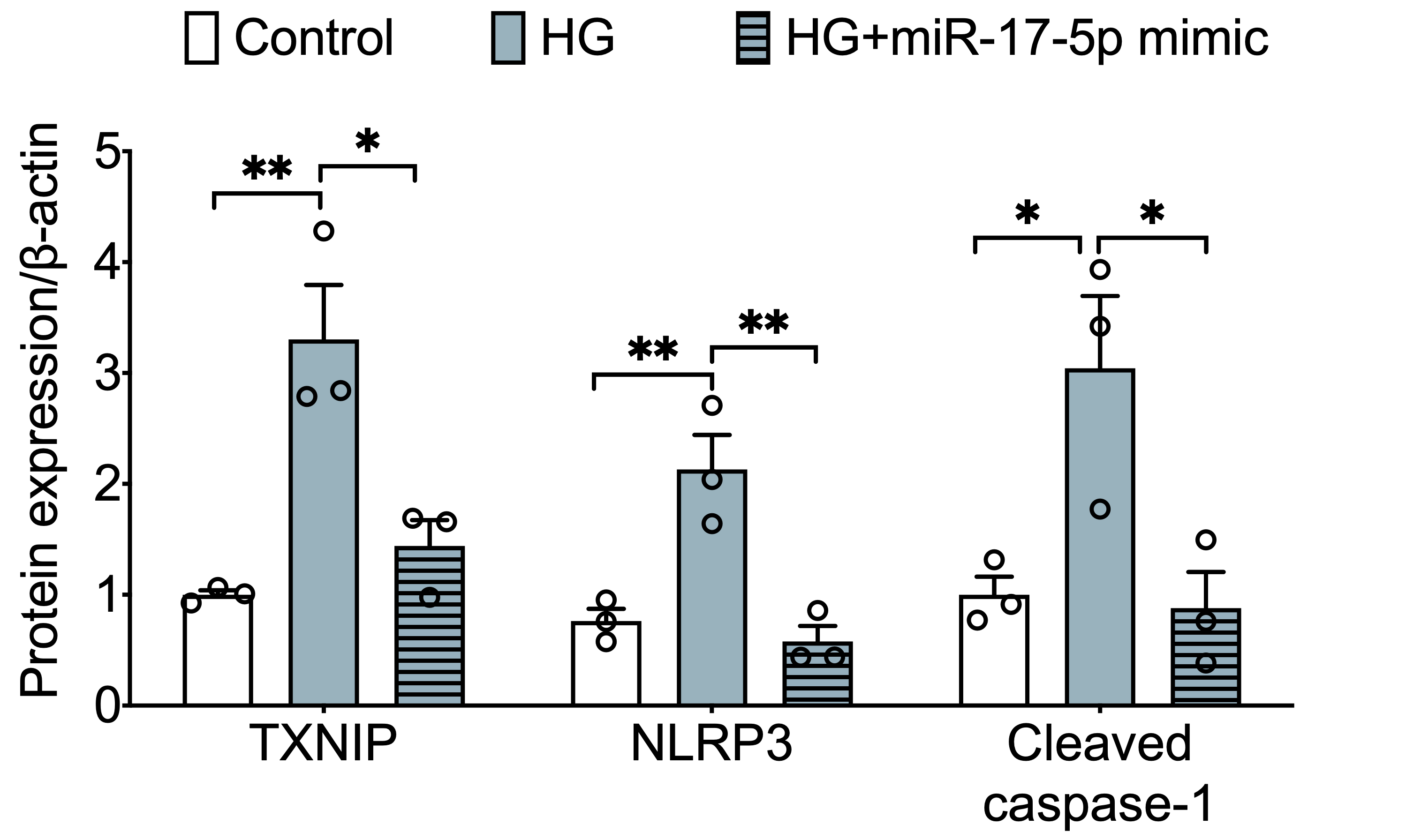

Supplement: Supplementary file 2 [file Data_Sheet_2.ZIP › data8.30/figure2-3/figure2ab-3abstatistics/figure2b.tiff]

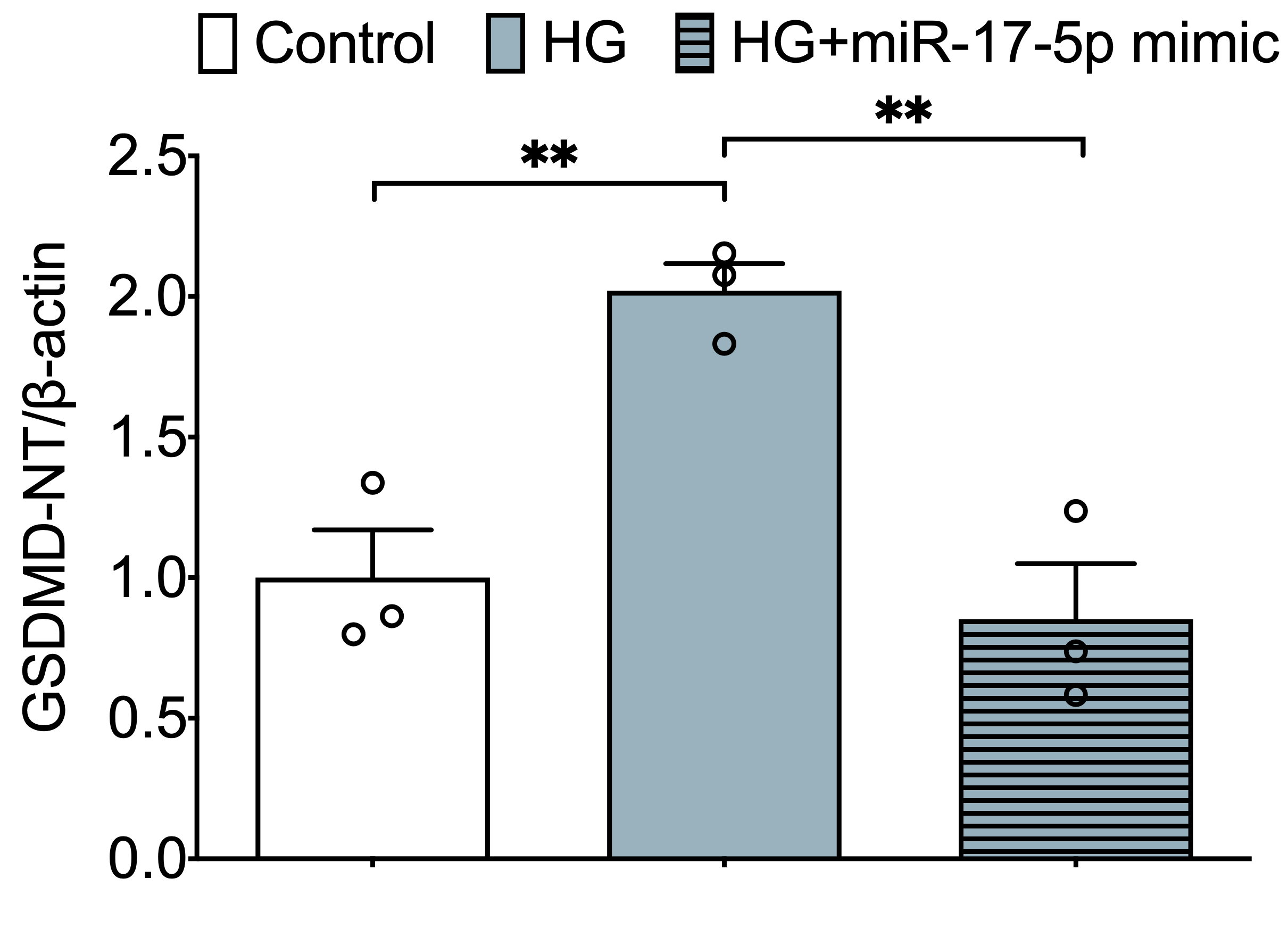

Supplement: Supplementary file 2 [file Data_Sheet_2.ZIP › data8.30/figure2-3/figure2ab-3abstatistics/figure3b.tiff]

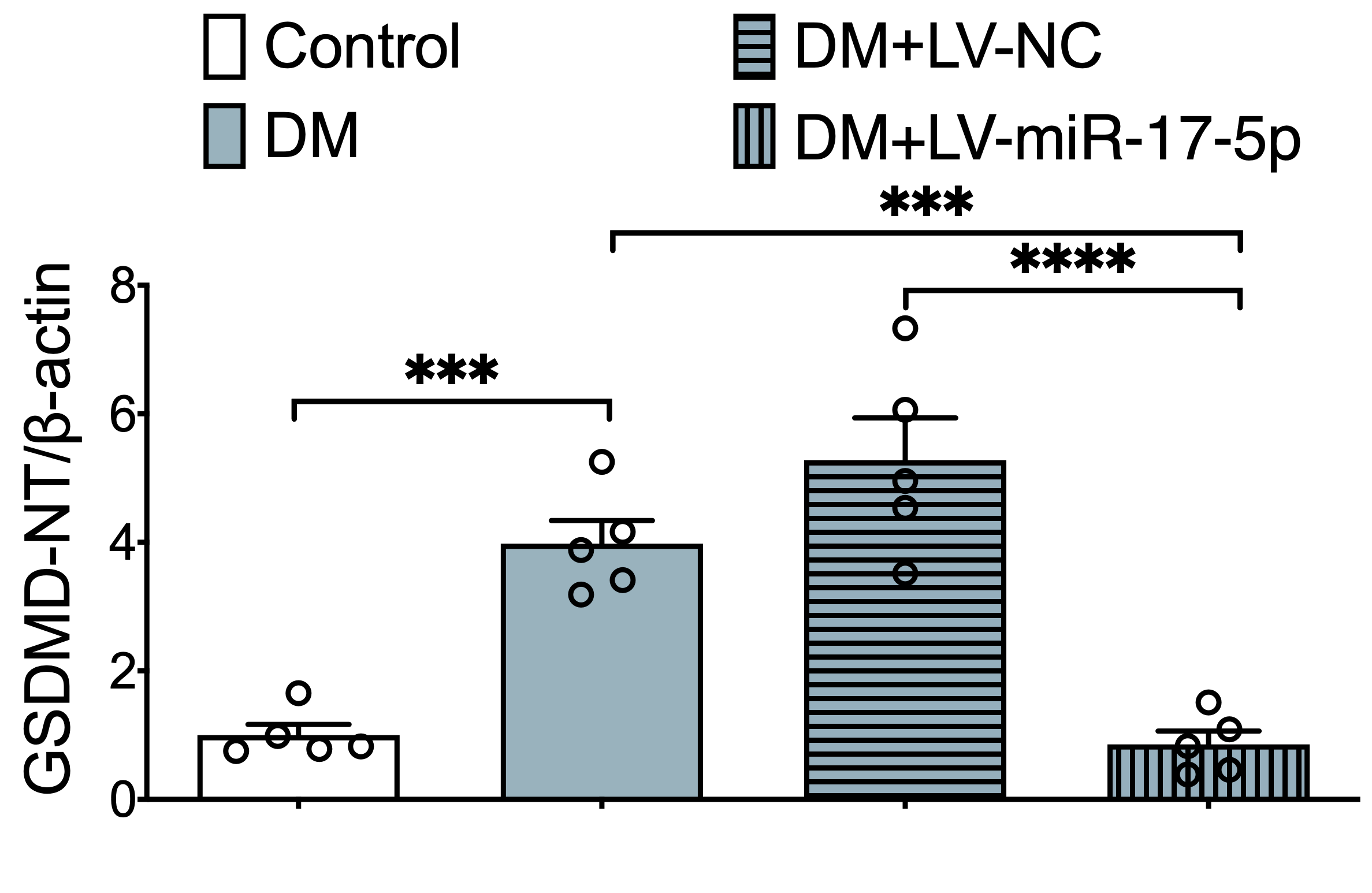

Supplement: Supplementary file 2 [file Data_Sheet_2.ZIP › data8.30/figure 5/statistics/figure5d.tiff]

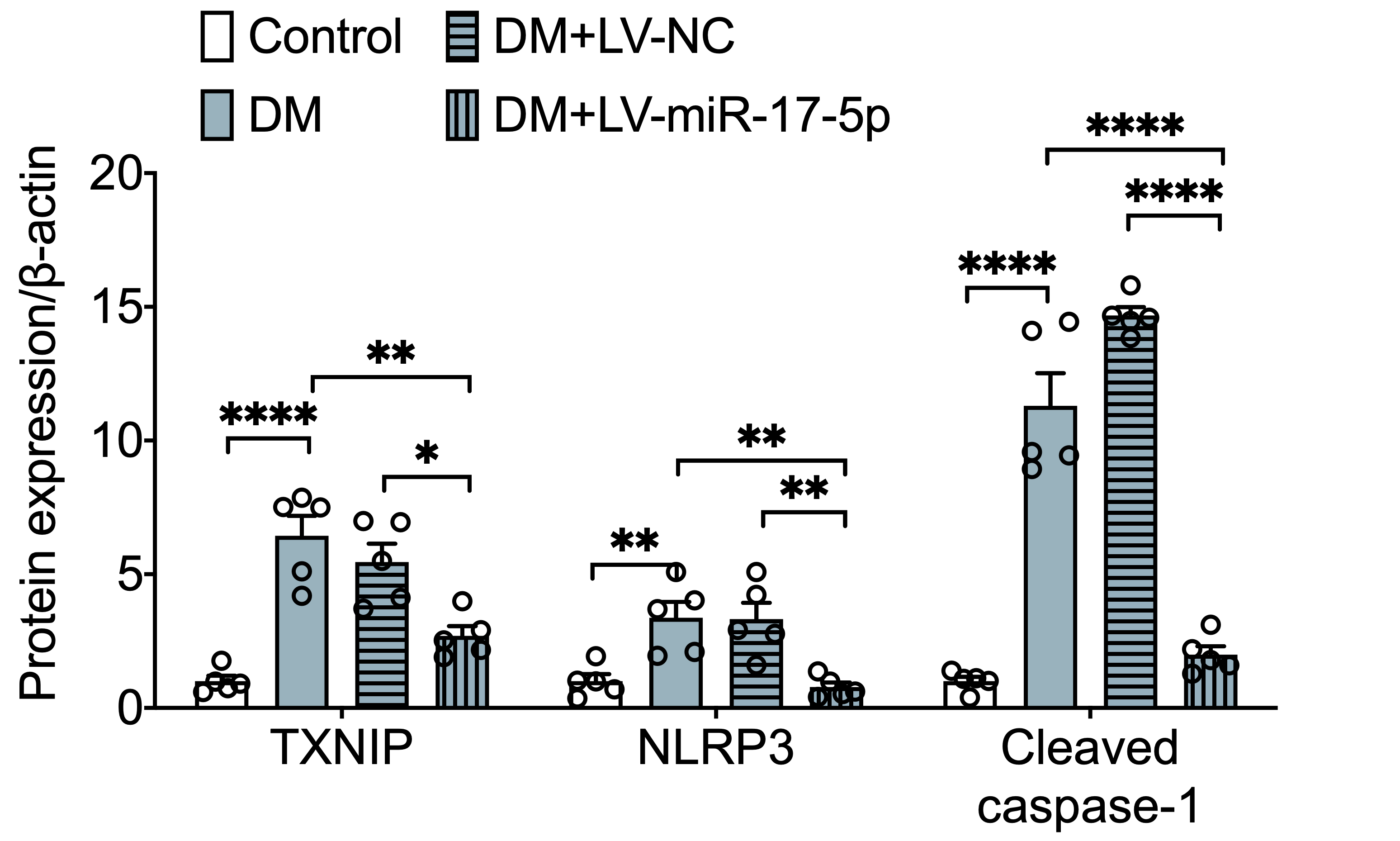

Supplement: Supplementary file 2 [file Data_Sheet_2.ZIP › data8.30/figure 5/statistics/figure5b.tiff]

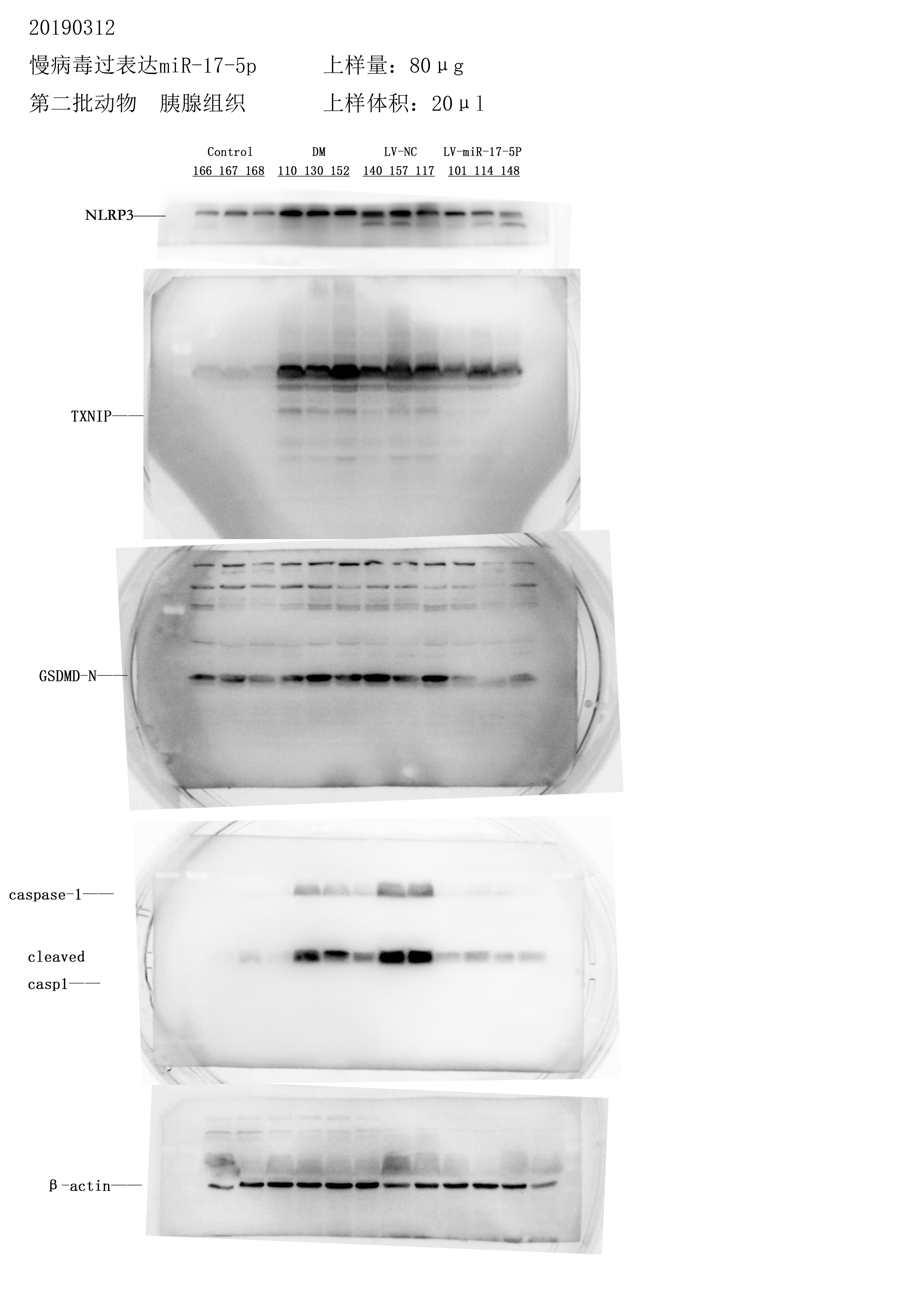

Supplement: Supplementary file 2 [file Data_Sheet_2.ZIP › data8.30/figure 5/picture/figure5ab-2.tif]

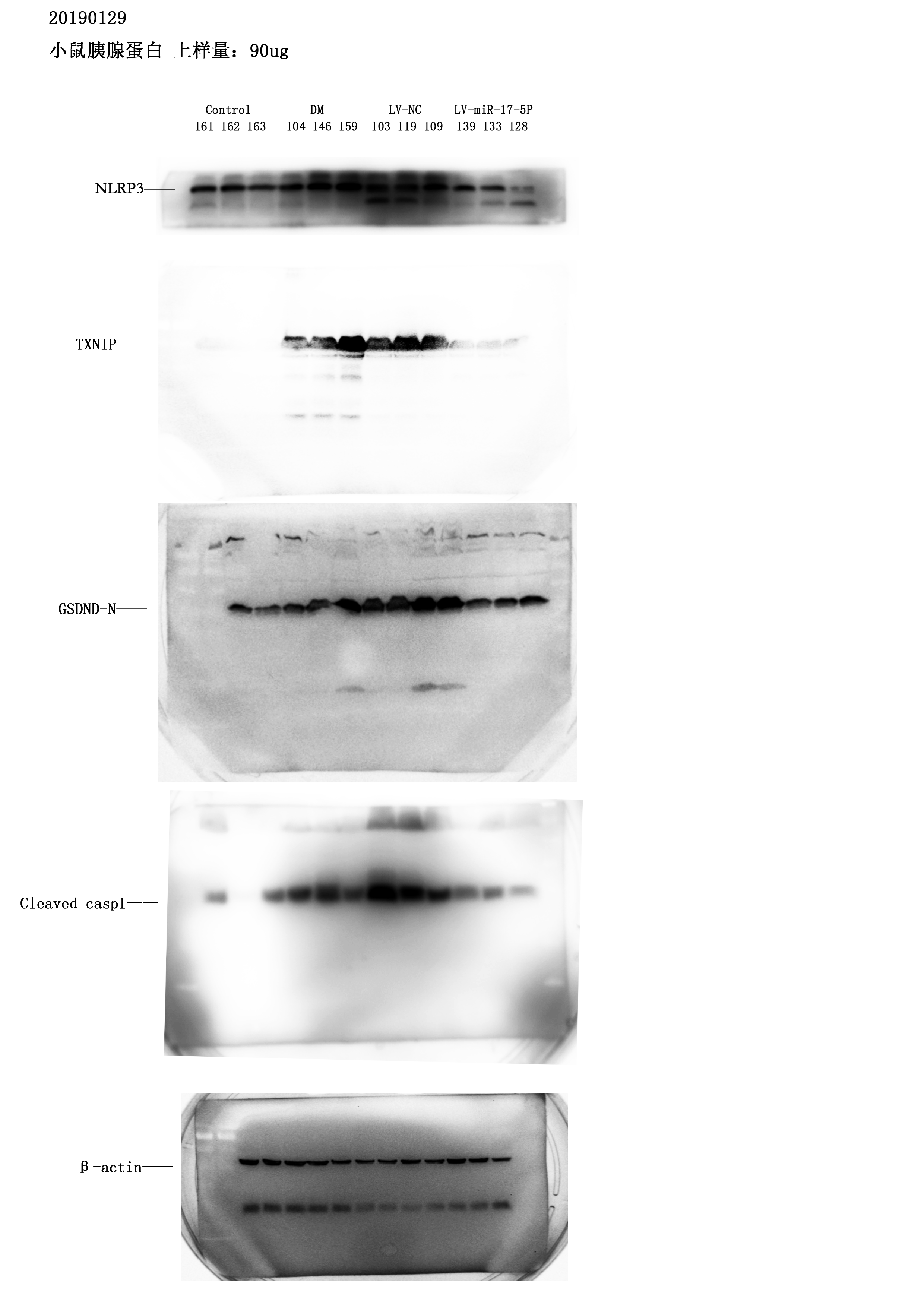

Supplement: Supplementary file 2 [file Data_Sheet_2.ZIP › data8.30/figure 5/picture/figure5ab-a.tif]
