# Supplementary material for: MiR-17-5p Inhibits TXNIP/NLRP3 Inflammasome Pathway and Suppresses Pancreatic β-Cell Pyroptosis in Diabetic Mice
Source: Front Cardiovasc Med. 2021 Nov 22;8:768029. doi: 10.3389/fcvm.2021.768029 (PMC8645844; doi:10.3389/fcvm.2021.768029)
Supplement: Supplementary file 2 [file Data_Sheet_2.ZIP › data8.30/figure1/figure 1CD/picture/figure 1C.pdf]

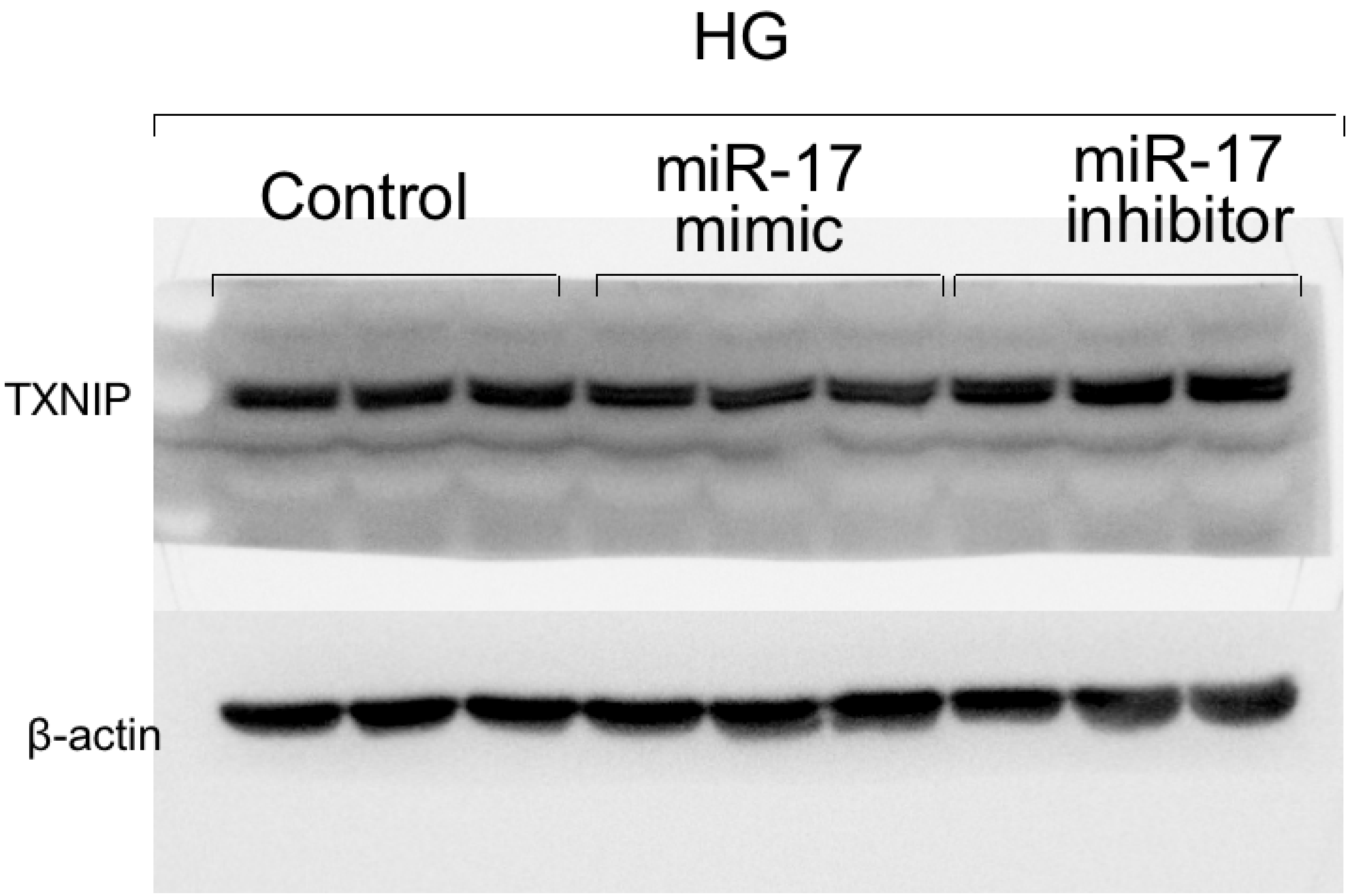

HG
Control
miR-17 mimic
miR-17 inhibitor
TXNIP
β-actin
